# Protection against Shiga Toxins

**DOI:** 10.3390/toxins9020044

**Published:** 2017-02-03

**Authors:** Simona Kavaliauskiene, Anne Berit Dyve Lingelem, Tore Skotland, Kirsten Sandvig

**Affiliations:** 1 Department of Molecular Cell Biology, Institute for Cancer Research, Oslo University Hospital, N-0379 Oslo, Norway; simona.kavaliauskiene@rr-research.no (S.K.); Anne.Berit.Dyve@rr-research.no (A.B.D.L.) Tore.Skotland@rr-research.no (T.S.); 2 Center for Cancer Biomedicine, Faculty of Medicine, Oslo University Hospital, N-0379 Oslo, Norway; 3 Department of Biosciences, University of Oslo, N-0316 Oslo, Norway

**Keywords:** Shiga toxin, Stx1, Stx2, hemolytic uremic syndrome, inhibitors, chloroquine, fluorodeoxyglucose, Mn^2+^

## Abstract

Shiga toxins consist of an A-moiety and five B-moieties able to bind the neutral glycosphingolipid globotriaosylceramide (Gb3) on the cell surface. To intoxicate cells efficiently, the toxin A-moiety has to be cleaved by furin and transported retrogradely to the Golgi apparatus and to the endoplasmic reticulum. The enzymatically active part of the A-moiety is then translocated to the cytosol, where it inhibits protein synthesis and in some cell types induces apoptosis. Protection of cells can be provided either by inhibiting binding of the toxin to cells or by interfering with any of the subsequent steps required for its toxic effect. In this article we provide a brief overview of the interaction of Shiga toxins with cells, describe some compounds and conditions found to protect cells against Shiga toxins, and discuss whether they might also provide protection in animals and humans.

## 1. Introduction

Shiga toxins (Stxs) comprise a family of related bacterial protein toxins that are similar in structure and mechanism of action, but are produced by different types of bacteria. Shiga toxin is secreted by *Shigella dysenteriae*, whereas Shiga-like toxin 1 (Stx1) and Shiga-like toxin 2 (Stx2) are produced by certain strains of *Escherichia coli* (Shiga toxin-producing *E. coli* (STEC)) and some other bacteria [1]. Prototypic Stx1 (Stx1a) differs from Shiga toxin only in one amino acid residue in the catalytic A-moiety of the toxin, whereas Stx2 shares only ~60% sequence similarity with Shiga toxin and defines an immunologically distinct subgroup comprised of at least seven subtypes of Stx2 [2]. Stx2 is more lethal than Stx1 in animal models [3,4] and is thought to be the main cause of life-threatening infections in humans. Some STEC produce only one toxin type, either Stx1 or Stx2, while others express a combination of both types and different subtypes [5]. For simplicity, we will use the abbreviation Stx to refer to the whole family of Shiga toxins when discussing general facts about the toxin and/or where the exact type or variant is not known. 

Infection with enterohemorrhagic STEC may cause hemorrhagic colitis, hemolytic uremic syndrome (HUS), and death [6]. There is no approved treatment of STEC-induced HUS, and the use of antibiotics may worsen the disease by increasing toxin formation and release by the bacteria [7]. In general, HUS occurs in 5%–15% of cases with STEC infection, with children having the highest risk [8], although the large outbreak with a Stx2a-producing enteroaggregative STEC strain in Northern Europe in 2011 demonstrated that there are bacterium-toxin combinations that can be as dangerous to adults as to children [9]. HUS will most often occur 5–13 days after the onset of diarrhea, with a mortality of 3%–5% [10,11]. In addition to direct renal damage, neurological complications may also occur in HUS patients and are important determinants of severity of the condition and mortality rate [12,13,14,15]. Neurological symptoms may be caused by fatigue, cerebral microvascular thrombi, ischemia-hypoxia, or the direct neuronal effects of Stxs [12,14,16]. 

One of the first specific therapeutic approaches against infections with Stxs was the idea of sequestering the toxin once it is released in the gut. In this regard, a novel agent composed of silicon dioxide particles covalently linked to the trisaccharide moiety of the globotriaosylceramide molecule that mediates Stx binding (Synsorb^®^ Pk, Synsorb Biotech) was developed. However, although Synsorb^®^ Pk was shown to bind and neutralize Stx1 (and Stx2, but less efficiently) in vitro [17], it failed to improve the clinical course of diarrhea-associated HUS in pediatric patients when tested in a randomized clinical trial [18]. The main drawback of neutralization of Stxs in the intestine for the prevention of HUS is that only trace amounts of the toxin reaching circulation are sufficient to induce HUS, and thus a more systemic treatment is required. Taking this into account, analogues of the globotriaosylceramide (Gb3) receptor and Stx antibodies for systematic administration have been developed and proven promising in in vivo models [19,20,21]. In addition, human serum amyloid component P (HuSAP) has been found to neutralize Stx2, but not Stx1, in vitro [22], and to protect mice against a lethal dose of Stx2 [23]. Moreover, eculizumab, an antibody directed against the complement protein C5, was used in patients with HUS during the outbreak in Northern Europe in 2011 [24] in order to counteract the activation of complement by the toxin [25]. These novel strategies based on direct neutralization of Stx in the intestine and/or circulation and the inhibition of complement have been well described in a recent review by Melton-Celsa and O’Brien [26] and thus are not further discussed here. In this review we will first provide a short overview of the toxin structure, toxin binding to the glycosphingolipid Gb3, and the intracellular transport, before we focus on the potential therapeutic agents for treatment of STEC infections and HUS that target specific cellular functions and protect cells against Stx by inhibiting toxin binding and/or intracellular trafficking.

### 1.1. Stx Structure

**Figure 1:**
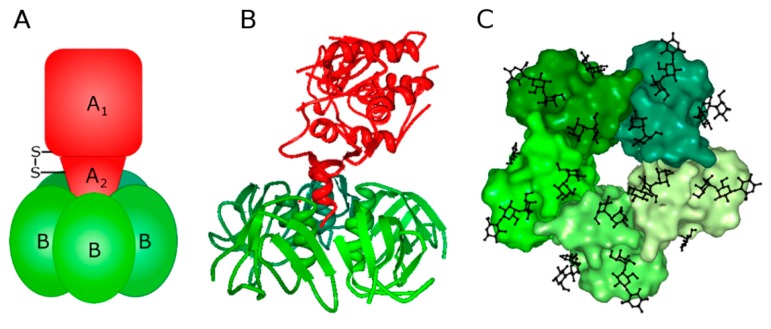
The schematic and structural models of Shiga toxins (Stxs). (**A**) Stxs consist of two non-covalently linked moieties: an A-moiety of ~32 kDa (shown in red), and a B-moiety (shown in green), comprised of five 7.7 kDa B-chains [27,28]. During intracellular toxin transport, the A-moiety is cleaved by the protease furin [39] into two fragments: an enzymatically active A_1_ fragment (~27 kDa) and a carboxyl terminal A_2_ fragment, which remain linked by a disulfide bond until arrival to the endoplasmic reticulum (ER) [40]. (**B**) The structure of the holotoxin as determined by *X*-ray crystallography [28] (PDB ID:1DM0); (**C**) The receptor-binding surface of the B-pentamer based on the structure of Stx1 complexed with the Gb3 analogue MCO-PK (methoxycarbonyloctyl glycoside of P^k^ trisaccharide) [33] (PDB ID:1BOS); the sugar moieties of MCO-PK are shown in black. Structure images were prepared using PDB ProteinWorkshop 4.2.

Stxs belong to the AB_5_ class of protein toxins and consist of an A-moiety (~32 kDa), which is non-covalently attached to a homo-pentameric B-moiety (7.7 kDa per monomer) (Figure 1) [27,28]. Nearly all Stxs bind exclusively to the globotriaosylceramide Gb3 [29,30,31] with the exception of one Stx2 subtype, Stx2e, which has been shown to bind to Gb4 [32]. Each B subunit harbors three Gb3 binding sites [33], making the toxin capable of binding up to 15 Gb3 molecules on the cell surface (Figure 1C). However, not all binding sites have equal affinity for the carbohydrates of Gb3 [34,35] and, therefore, not all sites might be required for binding to the cell surface, but might rather mediate additional recognition. The B-moiety alone is not toxic to cells (with the exception of B cells, where it may induce apoptosis [36]) and functions as a delivery tool for the enzymatically active A-moiety. It is still not clear why Stx2 is more lethal to humans than Stx1, but crystallographic studies and investigations of deletion mutants reveal important differences when it comes to the role of the C-terminal end of the A-subunit for retrograde transport and complex stability [28,37,38].

### 1.2. Gb3 and Its Interaction with Stx

**Figure 2:**
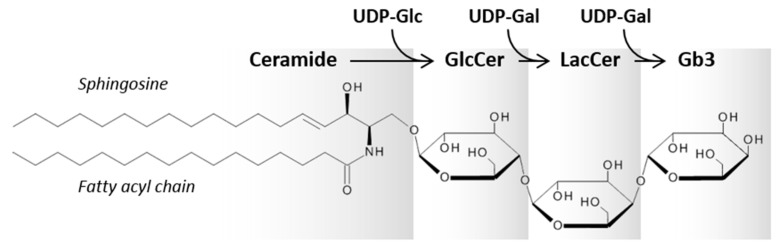
Chemical structure and biosynthesis of the Stx receptor globotriaosylceramide (Gb3). Sphingosine most often contains 18 carbon atoms, whereas the fatty acyl chain of ceramide varies both in length and saturation (here shown as C16:0). Gb3 is synthesized from LacCer by the addition of one galactose, and the reaction is catalyzed by Gb3 synthase (lactosylceramide α-1,4-galactosyltransferase). The sugar chain for Gb3 is: Gal-α1→4Gal-β1→4Glc-β1→Ceramide.

Globotriaosylceramide (Gal-α1→4Gal-β1→4Glc-β1→Cer, Gb3; Figure 2) is a glycosphingolipid expressed on the surface of certain cell types. Gb3 is formed by the addition of one galactose residue to lactosylceramide (LacCer), which is a common precursor for different classes of glycosphingolipids, and the reaction is catalyzed by Gb3 synthase (lactosylceramide α-1,4-galactosyltransferase). Gb3 is the first glycosphingolipid in the globo-series and thus serves as a precursor for the synthesis of more complex globo-series glycosphingolipids, such as globotetraosylceramide (Gb4). Gb4 is formed after addition of *N*-acetylgalactosamine (GalNAc) to the terminal galactose of Gb3. Although Gb3 is the primary receptor for all Stxs, it has been suggested that Gb4 might facilitate Stx2 binding to colon epithelium cells, which normally have no Gb3 or very low levels of Gb3 [41].

The sphingosine chain in the ceramide part of Gb3 is relatively invariable (most often it is monounsaturated with 18 carbon atoms, i.e., d18:1), but the *N*-amidated fatty acyl chain varies both in length (most common are 16–24 carbon atoms) and saturation resulting in multiple Gb3 species present in cells. Importantly, the receptor function of Gb3 has been shown to depend on its species composition [42,43,44,45], which in turn depends on cell type [46] and growth conditions [47], and might change in response to certain treatments, like exposure to butyric acid and cytokines [43,48,49,50,51,52]. It has been suggested that the production of butyric acid by the bacterial flora in the intestine may affect the expression and composition of Gb3 in the target cells and in turn lead to different susceptibility to the toxin between individuals [53]. In addition, the turnover time of Gb3 in the cells depends on its species, with longer fatty acyl chain-containing species having a longer half-life than the species with short fatty acyl chain [54]. Thus, inhibition of Gb3 synthesis will primarily lead to changes in Gb3 species composition in cells [43,50]. Studies based on artificial systems, where Gb3 was immobilized on thin layer chromatography (TLC) or ELISA plates, have shown that Stx1 and Stx2 have different binding preferences for different Gb3 species [44,55], although a mixture of various Gb3 species was required for the highest binding affinity [45]. Stx1 binding to Gb3 has also been shown to depend on cholesterol levels in the membrane [56,57]. Stx2 has been shown to be more potent in mice [3] and is more often associated with disease in humans [58], although the binding affinity of Stx2 to Gb3 is lower than that of Stx1, when measured using Gb3 adsorbed on a microtiter plate [59], and Stx2 is less toxic to Vero cells than Stx1 [60]. The different pathology observed for Stx1 and Stx2 might be caused by differences in receptor binding and thus differential targeting to susceptible tissues [61,62,63], as well as differences in intracellular transport of the toxins [37,64,65,66].

In the human body, the expression of Gb3 is restricted to only certain tissues. Normally, the highest Gb3 content is found in the microvascular glomeruli and proximal tubule cells of the kidney, consistent with the renal pathology of HUS [63,67,68,69]. Gb3 is also found in microvascular endothelial cells, and during infection with STEC, main Stx-target sites are the vascular endothelium of the colon [70,71] and the central nervous system [16,72]. Moreover, Gb3 is expressed in platelets [73,74], and in the carbohydrate defined P histo-blood group system, Gb3 constitutes the rare P^k^ antigen present on erythrocytes [75]. In the immune system, Gb3 represents a lymphocyte differentiation antigen, termed CD77, which is expressed in a subset of germinal center B lymphocytes [76]. It should also be noted that Gb3 expression is frequently increased in cancer cells [77]. However, the physiological role of Gb3 is still unclear, and it is not known why Gb3 expression is restricted to certain tissues. In vivo studies of Gb3 synthase knock-out mice, which displayed a total loss of Gb3 and other globo-series glycosphingolipids, showed no changes in birth-rates and no apparent abnormalities over a year of nurturing, with the exception of total loss of sensitivity to Stx1 and Stx2 as compared to wild-type mice [78].

### 1.3. Intracellular Transport of Stx

Upon binding to cells, the toxin has been found to activate a number of tyrosine kinases, including Syk [79], and the Src kinases Yes [80] and Lyn [81], as well as the serine/threonine protein kinase Cδ (PKCδ) [82] and the mitogen-activated protein kinase (MAPK) p38α [83]. Although the exact mechanism of how Stx mediates these signaling events is not yet fully understood, a recent study from our group have shown that the activation of Syk depends on the multivalent cross-linking of Gb3 at the plasma membrane, which in turn leads to an increase in cytosolic calcium levels and phosphorylation of Syk [84]. In addition, StxB binding to the cells has been shown to induce the release of cytoplasmic phospholipase A2 (cPLA2) from a cPLA2-annexin A2 complex, thereby facilitating Golgi transport, which has been found to be dependent on cPLA2 [85]. Furthermore, binding of the Stx B-moiety has been reported to stimulate remodeling of cytoskeleton components, such as actin, ezrin and dynein [86,87,88]. Thus, Stx is able to induce cell signaling and to modulate various cellular components to favor its uptake and intracellular transport.

**Figure 3:**
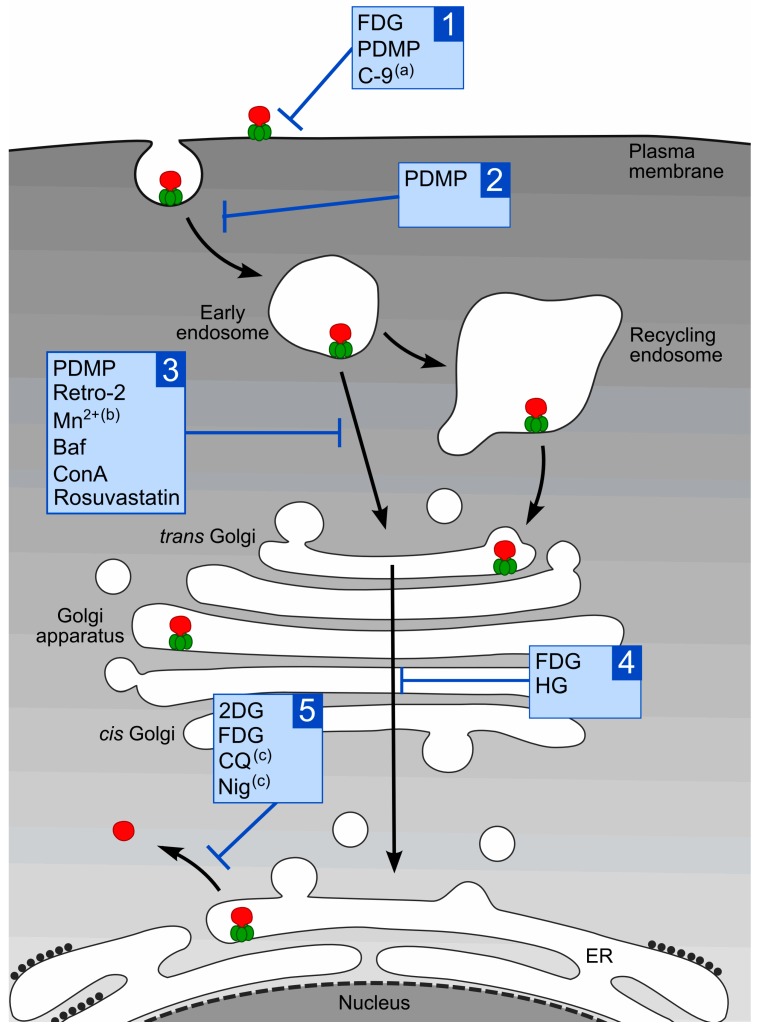
Stx uptake and intracellular transport, and the steps affected by different compounds. Stx binds to Gb3 on the cell surface and is taken up by various endocytic mechanisms. Following endocytosis, the toxin is transported through early endosomes and recycling endosomes and to the Golgi apparatus. From the Golgi, Stx is transported retrogradely to the ER, where its catalytically active A_1_-subunit is released and translocated into the cytosol. The different compounds discussed in this review are shown with their suggested action on different steps of Stx intoxication: 1—Stx binding; 2—Stx endocytosis; 3—Stx sorting to the Golgi; 4—Stx transport via Golgi to ER; 5—release and translocation of StxA_1_; (a) predicted effect for Stx2; (b) no effect for Stx2; (c) predicted effect for Shiga toxin.

Receptor-bound Stx becomes internalized by different endocytic mechanisms, including both clathrin- and dynamin-dependent and independent pathways [1]. After internalization, the toxin is transported from early/recycling endosomes directly to the Golgi apparatus [89] and then further to the endoplasmic reticulum (ER) [90,91,92,93]. During the transport, the A-moiety is cleaved by the protease furin, leaving two fragments, A_1_ and A_2_ (Figure 1), which remain linked to each other by a disulfide bridge [39]. Cleavage of Stx is optimal at low pH [94], indicating that it can occur early in the transport pathway. However, cells that lack furin can also cleave Stx, but less efficiently and at a later stage of the transport [39,95]. In the ER, the disulfide bond between the A_1_ and A_2_ subunits is reduced and the A_1_-subunit is released from the toxin. Finally, the A_1_-subunit is translocated across the ER membrane and inhibits protein synthesis by cleaving one adenine residue from the 28S RNA of the 60S ribosomal subunit [1]. However, the action of Stx in the cells is not limited to the inhibition of protein synthesis, and other cellular responses, such as cytokine expression and apoptosis, are triggered by the toxin (for review see [96,97]). Thus, efficient intracellular Stx transport depends on various cellular proteins and kinases, ER chaperones and other factors (for review see [1]). Drugs that affect any of these factors might interfere with proper intracellular transport of Stx and protect cells against the cytotoxic action of the toxin. In the next sections we will give an overview of compounds shown to protect cells against Stx, and we will discuss mechanisms responsible for the protection against the toxin, as well as the potential applicability of these drugs in vivo. An overview of the compounds discussed here and how they might act against Stx is given in Table 1. The intracellular transport of Stx and which steps of the transport are affected by the different compounds are shown in Figure 3. 

## 2. Compounds that Protect Cells against Stx

### 2.1. Chloroquine

Chloroquine (CQ; *N*′-(7-chloroquinolin-4-yl)-*N*,*N*-dietyl-pentane-1,4-diamine) is a weak base that in its unprotonated form easily can diffuse across membranes and accumulate in acidic compartments of the cell. There, CQ becomes protonated and trapped, leading to an elevated pH and swelling of the compartments. CQ (first named resochin) was developed in 1934 by Bayer laboratories as a synthetic antimalarial drug [113]. CQ was FDA-approved in 1949 and has proven to be one of the most effective and best tolerated agents against malaria [113,114]. It was widely used throughout the world in the 1950s and 1960s, and also later, but due to the emergence of the CQ-resistant malaria parasites *Plasmodium falciparum* and *Plasmodium vivax*, CQ has been abandoned as a prophylactic drug in most countries [115]. CQ and its analogues are also FDA-approved for the treatment of systemic lupus erythematosus and rheumatoid arthritis, and are in clinical trials as an adjuvant for anti-cancer chemotherapy and radiotherapy [115,116].

In short-term 3 h toxicity experiments in HEp-2 cells, a 2 h preincubation with 100 µM CQ gave a 15-fold increase in the Shiga toxin concentration needed to inhibit protein synthesis by 50% (IC50) [98]. In the retrograde pathway, the acidification decreases in the direction from early endosomes to the Golgi and the ER, and one might therefore expect CQ to have the most prominent effect early in the retrograde pathway. However, Shiga toxin transport to the Golgi apparatus was not affected by CQ treatment, neither was release of the A_1_-moiety of the toxin in the ER, suggesting that transport to the ER was also normal [98]. Thus, CQ might interfere with StxA_1_ translocation into the cytosol. Other compounds that disrupt the pH gradient, such as the V-ATPase inhibitors bafilomycin A1 (Baf) and concanamycin A (ConA) and the ionophore nigericin (Nig), also protect against Shiga toxin [98]. In contrast to CQ, the V-ATPase inhibitors interfere with Shiga toxin transport to the Golgi [98]. Even though all the compounds inhibit acidification, they interfere with different toxin transport steps, suggesting that also pH-independent processes are affected. The V-ATPase inhibitors show poor selectivity in vivo [117,118], and are therefore not suitable for clinical application. 

Shiga toxin is shown to interact with the Sec61 channel [119,120], and the knockdown of Sec61B has been shown to protect cells against Shiga toxin [119], suggesting that the translocation of StxA_1_ into the cytosol is dependent on the Sec61 channel. Interestingly, CQ has previously been shown to block several channels, such as the inward rectifier potassium channel Kir2.1 [121], and the translocation pores of the pore-forming toxins anthrax and C2 toxin [122,123]. One can speculate that CQ might also interact with the Sec61 channel to block the translocation of Shiga toxin. Importantly, as shown here, CQ also protects against Stx2 (see below). 

**Figure 4:**
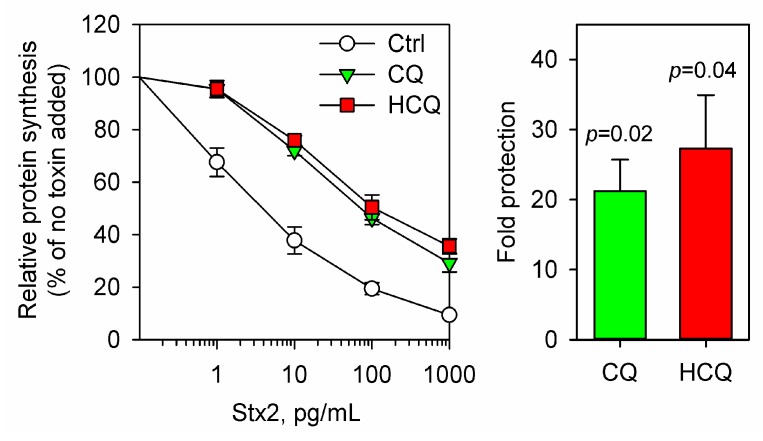
Cell protection against Stx2 by CQ and HCQ. HEp-2 cells were treated with or without 25 µM chloroquine (CQ) or 25 µM hydroxychloroquine (HCQ) in complete growth medium for 1 h prior to incubation with 10-fold serial dilutions of Stx2 for 24 h in the presence or absence of the drugs. The cells were then incubated in the presence of [^3^H]leucine for 20 min, and protein synthesis was measured as described in [98]. The left panel shows relative protein synthesis as a percentage of the samples without Stx2 added. The right panel shows relative fold protection against Stx2. The protection was calculated as an increase in IC50 for treated samples compared to control. The error bars show SEM (*n* = 4). One sample *t*-test was used for statistical analysis of the protection data, and obtained *p* values are given in the figure.

For acute treatment of malaria, the recommended dose of CQ is 10 mg/kg/day. CQ is rapidly distributed in the body and reaches a peak plasma concentration within 3–12 h after an oral dose [124]. In most cases, CQ is slowly eliminated with a half-life median value of 40 days, but there are big differences between individuals and the half-life of CQ has been reported to range from 1 to 157 days [124]. The plasma concentration is strongly dependent on the administered dose and the duration of the treatment [124]. The therapeutic concentration in blood of hydroxychloroquine (HCQ), a less toxic analogue of CQ with similar pharmacokinetics, has been reported to be between 0.03 and 15 mg/L; the toxic and lethal concentration ranges were 3–26 mg/L and 20–104 mg/L, respectively [125]. CQ at high concentrations has been reported to have a number of effects in cell cultures, but clinically safe and achievable doses are in the low micromolar range [116]. Thus, it has been suggested that for clinical relevance, the CQ concentrations should not exceed 10 mg/L or 31 µM [116]. As shown in Figure 4, in long-term toxicity experiments (24 h with toxin), a 1 h preincubation with a concentration of 25 µM CQ gave approx. 20 fold protection against Stx2, indicating that CQ doses within the therapeutic window might be sufficient to protect against the toxin. HCQ also protected cells against Stx2 to a similar degree as CQ (Figure 4).

It should be noted that in animal models, CQ was shown to strongly accumulate in tissues, for instance in the uvea, liver, lungs, and kidneys [124]. A similar distribution has been reported in humans [124]. Thus, the CQ concentration in the target cells of Stx might be a lot higher than the plasma concentration.

### 2.2. The Glucose Analogues 2-Deoxy-d-glucose and 2-Fluoro-2-deoxy-d-glucose

2-Deoxy-d-glucose (2DG) is a structural analogue of glucose, and has been used in research as a glycolytic inhibitor since 1950s [126,127]. 2DG differs from glucose only by the absence of one oxygen atom at the second carbon. Similarly to glucose, 2DG is taken up through the glucose transporters and phosphorylated by hexokinase to form 2DG-6-phosphate (2DG-6-P). However, 2DG-6-P is not metabolized further and accumulates in the cells [127,128,129]. 2DG-6-P inhibits glycolysis by competing with glucose-6-P for phosphoglucose isomerase [127], and by acting as a non-competitive inhibitor of hexokinase [130]. However, although the inhibition of glycolysis has been a commonly exploited effect of 2DG, this compound has a much broader spectrum of activities. In addition to inhibiting glycolysis, 2DG inhibits *N*-linked protein glycosylation [131,132]. In turn, this leads to accumulation of misfolded proteins and triggers the unfolded protein response in the ER, leading to ER stress [133,134]. Interestingly, Okuda et al. recently discovered that 2DG inhibits the expression of the Gb3 synthase by yet unknown mechanisms, and thus reduces cellular Gb3 levels in the cells [135]. 

2-Fluoro-2-deoxy-d-glucose (FDG) is a structural analogue of glucose where the hydroxyl group at the second carbon is replaced by a fluorine atom. Like glucose and 2DG, FDG is transported into cells, where it is phosphorylated by hexokinase to yield FDG-6-P. However, FDG-6-P does not undergo isomerization to fructose and thus cannot be further catabolized, leading to accumulation of FDG-6-P in the cells [136]. Similarly to 2DG, FDG also inhibits glycolysis, but because the binding energy of FDG-6-P for the allosteric site of the hexokinase is lower than that of 2DG-6-P, and closely resembles the energy of glucose-6-P, FDG is a better inhibitor of glycolysis than 2DG [137]. FDG also interferes with *N*-linked protein glycosylation [131,138,139], but in contrast to 2DG, FDG does not become incorporated into dolichol-linked oligosaccharides [138]. FDG seems to slow down rather than prevent the assembly of the dolichol-linked oligosaccharide, and thus is a weaker inhibitor of *N*-glycosylation than 2DG. In addition, we have recently found that in contrast to 2DG, FDG does not become incorporated into newly synthesized glycolipids [48]. [^18^F]FDG, which contains the ^18^F radioisotope, is a commonly used imaging agent for positron emission tomography (PET). [^18^F]FDG based PET is widely used for diagnosis and monitoring of oncological, neurological, and cardiological diseases (for review see [140,141]). 

**Figure 5:**
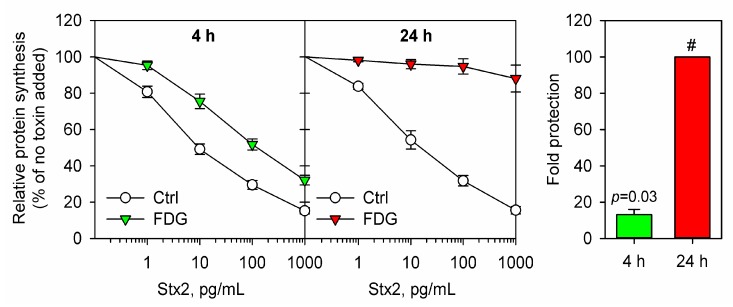
Cell protection against Stx2 by FDG. HEp-2 cells were treated with or without 1 mM 2-fluoro-2-deoxy-d-glucose (FDG) in complete growth medium for 4 h or 24 h prior to incubation with 10-fold serial dilutions of Stx2 for 24 h in the presence or absence of FDG. The cells were then incubated in the presence of [^3^H]leucine for 20 min, and protein synthesis was measured as described in [98]. The left panel shows relative protein synthesis as a percentage of the samples without Stx2 added. The right panel shows relative fold protection against Stx2. The protection was calculated as an increase in IC50 for treated samples compared to control. In the samples treated with FDG for 24 h, the highest toxin concentration tested (1 ng/mL) did not reduce protein synthesis down to 50%, therefore the fold-protection could not be calculated and was estimated to be more than 100-fold (marked as #). The error bars show SEM for 4 h treatment (*n* = 4) and the deviation from the mean of two independent experiments for 24 h treatment. One sample *t*-test was used for statistical analysis of the protection data for 4 h treatment, and the obtained *p* value is given in the figure.

We have recently discovered that both 2DG and FDG reduce cell sensitivity to both Shiga toxin and Stx2. Four hours preincubation with either 10 mM 2DG or 1 mM FDG led to an increase in the IC50 for Shiga toxin by 13-fold in HEp-2 cells [48,99]. In addition, 24 h preincubation with 10 mM 2DG reduced cell sensitivity to Shiga toxin by 30-fold [99], while 24 h pretreatment with 1 mM FDG made HEp-2 cells fully resistant to both Shiga toxin and Stx2 (maximal concentration tested was 100 ng/mL with 3 h challenge) [48]. For FDG, we have also tested whether the protective effect is observed in non-cancer originated cells, and found that immortalized human brain microvascular cells (HBMEC) also became less sensitive to Shiga toxin after 4 h and 24 h pretreatment with 1 mM FDG [48]. However, all these toxicity assays have been performed with a relatively short 3 h challenge with the toxin. Thus, we have now also tested whether FDG protects HEp-2 cells against 24 h challenge with Stx2, and observed an essentially similar protection as shown for 3 h incubation with the toxin (Figure 5).

**Figure 6:**
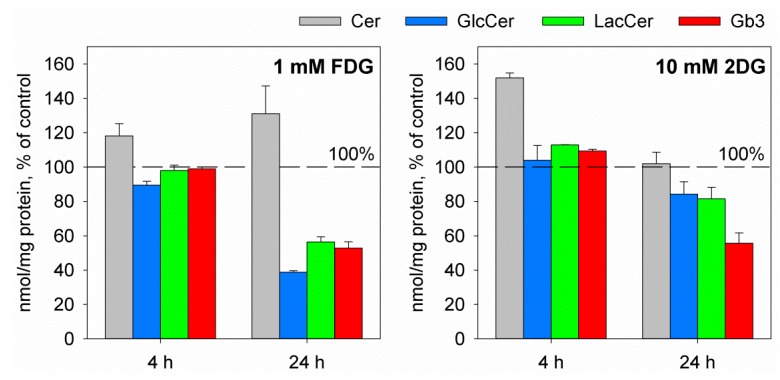
Effect of FDG and 2DG on total levels of Gb3 and its precursors. Cells were treated with or without 1 mM FDG or 10 mM 2DG for 4 h or 24 h, and lipids were analyzed by mass spectrometry in whole-cell lysates. The total amount of lipid was normalized to the total amount of protein in each sample (protein content was measured by BCA assay). The graph shows the levels of Cer, GlcCer, LacCer, and Gb3 in treated cells compared to control samples; the error bars show the deviation from the mean of two biological samples. For a detailed method description and the raw data see [48,99].

Interestingly, although both 2DG and FDG were found to reduce cellular Gb3 levels by 50% following 24 h treatment (Figure 6), it was only FDG that also led to reduction in Shiga toxin binding [48,99]. The mechanisms by which 2DG and FDG inhibit Gb3 synthesis are still not clear and seem to be different, as 2DG has been shown to inhibit the transcription of the Gb3 synthase gene [99,135], while FDG has no effect on the expression of Gb3 synthase [48]. In addition, we have found that cell treatment with FDG also reduces cellular levels of LacCer and glucosylceramide (GlcCer) (Figure 6) [48], indicating that FDG inhibits the synthesis of GlcCer and thus depletes cells for the precursors required for Gb3 synthesis, rather than inhibiting the synthesis of Gb3 directly.

Although the inhibition of Gb3 synthesis might be an important factor for the protection against Stx by 2DG and FDG after long-term treatment, the protection observed after 4 h treatment with drugs does not seem to be mediated by changes in Gb3 [48,99]. We have shown that following 4 h treatment with either 10 mM 2DG or 1 mM FDG the intracellular transport of Shiga toxin is changed and most likely accounts for the protection observed at this time point. Four hours pretreatment with 10 mM 2DG almost completely blocked the release of Shiga toxin A_1_-moeity in the ER in HEp-2 cells, which correlated well with the depletion of calcium from the ER [99]. Although it has been proposed that 2DG induces release of calcium from the ER via induction of ER stress [142], combined treatment with mannose, which rescues 2DG-mediated ER stress [143], does not prevent calcium leakage from the ER upon 2DG treatment and does not rescue cell sensitivity to Shiga toxin [99]. Three ER chaperones, HEDJ (also called ERdj3), BiP (also called GRP78) and GRP94 (glucose-regulated protein of 94 kDa), have been shown to bind the A-moiety of Stx1 [120], and thus are suggested to be involved in the release of StxA_1_ in the ER. Substrate binding to GRP94 and BiP has been shown to be regulated by calcium [144,145], suggesting that 2DG might prevent the release of StxA_1_ in the ER by inhibiting Shiga toxin interaction with chaperones. 

Similar to 2DG, FDG was found to deplete calcium from the ER and to inhibit release of the A_1_-moiety from the holotoxin [48]. However, since the concentrations for FDG and 2DG used were different, it is difficult to conclude whether the effect on ER calcium levels and the efficiency in blocking StxA_1_ release are similar for these drugs. However, we have found that FDG also inhibits Stx1 transport from the Golgi to ER [48], which did not seem to be the case for 2DG [99]. This additional block in the Stx transport might explain why FDG is more efficient than 2DG in protecting cells against Shiga toxin and Stx2 following short-term incubation. Furthermore, since the long-term preincubation (24 h) with FDG, but not 2DG, also leads to reduced Stx1 binding, it makes FDG a promising candidate drug for STEC infections. However, in vivo studies are required to test whether FDG could potentially be used for treatment. The first challenge is to reach high enough concentrations of FDG in the Stx-targeted cells. When [^18^F]FDG is used in the clinics for PET, the main limiting factor for the concentration of [^18^F]FDG used in patients is the allowed maximal radiation dose. This is not the problem when using stable nonradioactive FDG. There are no clinical studies describing maximal FDG doses that could be safely achieved in human plasma/tissues, but there are several clinical studies that have analyzed the safety of 2DG when used in combination with chemotherapy or radiation. Based on the study by Raez et al. [146] the recommended daily dose of 2DG in combination with docetaxel was 63 mg/kg, which resulted in a median maximum plasma 2DG concentration of 0.7 mM, and caused tolerable adverse effects. The tolerable concentration and adverse effects of FDG might differ from those shown for 2DG and have to be assessed in the future, but if the achievable concentration is similar to that shown for 2DG (0.7 mM), it is then in the same range as the concentration (1 mM) shown to protect cells against Stx in vitro [48].

### 2.3. Retro-2 Substances

By performing a high-throughput screening of 16,480 drugs Stechmann et al. [100] identified two low molecular weight substances that reduced sensitivity to Stx1, Stx2, and the plant toxin ricin (which also follows a retrograde route to the ER), when added 30 min prior to challenge with toxins in A459 and HeLa cells. These substances were called Retro-1 and Retro-2. They were reported to inhibit retrograde transport of Stx1B from endosomes to the Golgi apparatus without affecting compartment integrity and endogenous retrograde cargo transport [100]. Other compounds, which may have similarities to the Retro compounds, have also been shown to block trafficking and toxicity of Stx1 and ricin [147]. Both Retro-1 and Retro-2 have been found to relocalize the SNARE proteins syntaxin 5 and, to a lesser extent, syntaxin 6 from their normal site of accumulation on perinuclear Golgi membranes [100]. Thus, it has been suggested that the inhibition of Stx1B transport by these compounds could be mediated by the relocalization of syntaxin 5 and 6, although additional studies are required to confirm this. Retro-2 was found to be the most effective of these drugs, and an intraperitoneal injection of 200 mg/kg Retro-2 given 1 h prior to toxin challenge completely protected mice that were given a lethal nasal instillation of ricin (animal experiments with Stx were not performed) [100]. Later, this group and others published data for several substances similar to Retro-2, and showed that it was a cyclic form (Retro-2^cycl^) and not Retro-2 that was active [148,149,150]. Cyclization and modification of Retro-2 resulted in a compound with approx. 100-fold increased efficacy in inhibiting Stx1, and only one enantiomer was found to be active with an EC50 value (the concentration of the drug that gives 50% of its full inhibitory effect against the toxin) of approx. 300 nM in HeLa cells [149]. The most active of the Retro substances in counteracting the cytotoxicity of Stx1 (named (S)-Retro-2.1) has been reported to have an EC50 value of 54 nM in HeLa cells [151].

Secher et al. has investigated whether Retro-2^cycl^ could protect mice against the toxic effects of infection with *E. coli* O104:H4, the strain that was responsible for the deadly outbreak in Europe in 2011 [152,153]. The bacteria were given to mice by oral gavage, and on days 16 and 26 the mice received intraperitoneal injections of 100 mg/kg of Retro-2^cycl^ , which resulted in reduced mortality rate in the treated group (29 and 16 mice were dead in control and treated group, respectively, out of 40 mice per group) [101]. The same authors recently published a review article about the use of Retro-2 and similar compounds to protect against Stxs, ricin, multiple viruses, including different polyomaviruses, Ebola virus and poxviruses, and intracellular parasites, such as *Leishmania* [154], and we refer to this review for further discussions about these substances. The authors conclude that these lead compounds now need to be developed as drugs for human use. As these drugs, as far as we know, have not been given to humans or even been tested in formal preclinical drug safety studies, that task will be ongoing for many years. Furthermore, it will be interesting to see which compound will be selected for such a development as the most efficient compound may also be the most toxic. Finally, it should also be investigated whether all of these compounds have similar effects on the localization of syntaxin 5 as Retro-1 and Retro-2 [100], since targeting of a universal trafficking factor such as syntaxin 5 may prove challenging in a clinical setting.

### 2.4. Manganese

Mn^2+^-ions have been described by several groups to protect cells against Shiga toxin [105] and Stx1 [103]. Mukhopadhyay and Lindstedt reported that the protection was due to redirection of the toxin to the degradative pathway; they also reported that mice injected with a lethal dose of Stx1 could be rescued by a nontoxic dose of Mn^2+^ that was injected five days before the toxin [103]. However, Mn^2+^ failed to protect cells against Stx2 under the same conditions [65]. These authors also showed that elevated levels of Mn^2+^ resulted in a down-regulation of GPP130, which in turn led to inhibition of retrograde transport of Stx1, but not Stx2 [65]. Furthermore, this group later showed that the increased Mn^2+^ levels induced GPP130 oligomerization and its sorting to lysosomes for degradation [102]. 

The protective effect of Mn^2+^ against Stx toxicity was also studied by another group, and there the authors failed to see any protective effect of Mn^2+^ against Stx1 and Stx2 in cultured Vero cells and CD-1 outbred mice at Mn^2+^-doses that were not toxic to the cells and the animals [104]. They concluded that the ability of Mn^2+^ to protect against Stx toxicity might be dependent on the cell line and mouse strain, and that protection may be observed only at potentially toxic concentrations of Mn^2+^. These authors and others have discussed that Mn^2+^-ions are neurotoxic; for reviews see [155,156]. Due to this well-known toxic effect of Mn^2+^, we believe that it is unlikely that Mn^2+^ can be developed as a drug against Stx toxicity.

### 2.5. Inhibitors of GlcCer Synthesis PDMP and C-9 

The glycosphingolipid Gb3 is the sole functional receptor for Stx in humans, which makes it a potential target for preventing Stx toxicity to cell. The drawback of the approach directed towards the receptor is the time required to deplete Gb3 from cells, and this might limit the therapeutic potential. However, although the expression of Gb3 is a prerequisite for cell sensitivity to Stx, a specific Gb3 species composition [42,43,44,45] is required for efficient Stx binding and intracellular transport, meaning that a complete depletion of Gb3 might not be necessary to prevent Stx intoxication. To our knowledge, there are no compounds available that would specifically block Gb3 synthesis in the cells. However, multiple substances have been developed for inhibiting glucosylceramide synthesis, and thus prevent formation of more complex glycosphingolipids, including Gb3. Two such compounds, PDMP [43] and C-9 [106], have been shown to reduce cell sensitivity to Stx, and are discussed in this section. 

PDMP (1-phenyl-2-decanoyl-amino-3-morpholino-1-propanol) is a ceramide analogue first developed in a search for drugs to treat individuals with Gaucher disease [157], which are deficient of the lysosomal enzyme glucosylceramidase and thus accumulate GlcCer in certain tissues [158]. PDMP has been shown to inhibit the synthesis of GlcCer and specifically affect the content and composition of glycosphingolipids, without perturbing other lipid profiles [43,159], and without significantly affecting the synthesis of glycoproteins in cells [159]. Raa et al. showed that treatment with 1 µM PDMP for 24 h had only a small (20%–30%) effect on Shiga toxin binding to HEp-2 cells, but led to an approx. 50% reduction in the toxin uptake and almost completely blocked (by 90%) the transport of StxB into the Golgi [43]. These effects were accompanied by a 6.5-fold increase in the IC50 value for Shiga toxin in HEp-2 cells. After 24 h incubation with 1 µM PDMP, the cellular levels of Gb3 and its precursors were reduced by approx. 50% in treated HEp-2 cells, and the Gb3 species with the fatty acyl group 16:0 were found to be degraded faster and to a larger extent than the Gb3 with the fatty acid 24:1 [43]. Results from studies on butyric acid-mediated cell sensitization to Shiga toxin, and comparison of cell lines with different sensitivities to Stx, have indicated that Gb3 with certain fatty acyl groups might be important for endosome-to-Golgi transport [49,91,160,161]. Thus, the changes in Gb3 species composition may play an important role in the PDMP-induced protection against Stx, at least at shorter treatment times, when the total Gb3 is not yet completely depleted from the cells. However, in addition to GlcCer synthase, PDMP has been found to target other lipid enzymes, such as ceramide glycanase and a lysosomal phospholipase A2 called 1-O-acylceramide synthase [162,163], which might limit the applicability of PDMP for specific depletion of Gb3 in vivo. On the other hand, the development of PDMP has boosted the synthesis of a variety of related compounds [162]. Some of these PDMP analogues exhibit a dramatic increase in potency and specificity for the ceramide-specific glucosyl transferase [162], and have been tested in β-galactosidase a-null mice (model of Fabry disease in which Gb3 accumulates in the vasculature and kidneys) [164]. The mice were injected intraperitoneally with 2 mg/kg of the PDMP analogue EtDO-P4 (d-threo-1-(3,4-ethylenedioxyphenyl)-2-(palmitoylamino)-3-(1-pyrrolidinyl)propanol) twice a day for three days, which led to approx. 50% reduction in GlcCer levels in the kidney, liver, and heart. The reduction in Gb3 was little pronounced after three days of treatment, but following eight weeks of treatment with 10 mg/kg of EtDO-P4 twice a day, the total levels of Gb3 were reduced by approx. 50% in the kidney, liver and heart. Importantly, the treatment did not show apparent toxicity to the animals. However, a potential protection against Stx toxicity was not tested in this study. 

The search for treatment of glycosphingolipid storage diseases has led to the development of another ceramide analogue named C-9 [(1R, 2R)-nonanoic acid [2-(2′,3′-dihydro-benzo [1–4]dioxin-6′-yl)-2-hydroxy-1-pyrrolidin-1-ylmethyl-ethyl]-amide-L-tartaric acid salt] (Genzyme Corp., Waltham, MA, USA). Silberstein et al. has demonstrated that a 48 h pretreatment with 5 µM C-9 reduced cellular Gb3 levels by approx. 80% in human renal tubular epithelial cells (HRTEC), which was accompanied by an almost complete cell protection against a 24 h challenge with 1 ng/mL Stx2 [106]. However, the drug had no effect on the cell sensitivity to Stx2 when added at the same time as the toxin and a 24 h preincubation was required to obtain the protective effect [106]. Essentially similar protection and reduction in Gb3 was also shown in human glomerular endothelial cells [108]. Silberstein et al. also investigated the potency of C-9 against Stx2 in an in vivo model [107]. Rats received C-9 orally two days prior and four days after the intraperitoneal injection of the supernatant from recombinant *E. coli* expressing Stx2. The treatment reduced rat mortality by 50% and prevented intestinal and renal tissue damage, which was observed in the group treated with Stx2 only. The failure of C-9 to completely prevent rat mortality after Stx2 challenge was attributed to the possibility that C-9 does not pass the blood- brain barrier, and thus the deaths in the C-9 group could be the outcome of neurological injuries. However, no histological analysis was performed to support this [107]. 

A related compound, which differs from C-9 only in the fatty acid part (contains octanoic acid instead of nonanoic acid), has been approved by FDA for treatment of Gaucher disease [165] and is now sold under the name Cerdelga^®^ (Eliglustat tartrate; Genzyme Corp., Cambridge, MA, USA). Eliglustat tartrate (also called Genz-112638) is well tolerated and has a recommended dosing of 100 mg (contains 84 mg of eliglustat) twice daily in Gaucher patients [166,167]. When used at the recommended dosing, the average concentration of free eliglustat in the plasma is 12–25 ng/mL (but there is a great variation between individuals due to differences in the rate of metabolic degradation of eliglustat) (reviewed in [165]). Thus, it would be interesting to see whether eliglustat provides similar protection against Stx in cells and in animal models as C-9, and whether the tolerated dose leads to significant changes in Gb3 levels and/or species, which would indicate its potential use for the treatment of STEC infections. However, very little eliglustat is taken up into brain [164], which would limit its effect in the HUS cases with neuronal damage.

### 2.6. HG

The alkylglycerol 1-*O*-hexadecyl-*sn*-glycerol (HG, also called chimyl alcohol) is a precursor for ether-linked glycerophospholipids. HG enters the biosynthetic pathway of ether lipids after being phosphorylated by alkylglycerol kinase [168]. Addition of HG was shown to alter the lipidome of HEp-2 cells, with the most notable changes being an increase in the cellular level of ether-linked lipids with 16 carbon atoms at *sn*-1 position, an increase in lysophosphatidylinositol (LPI) and a decrease in all glycosphingolipid classes analyzed, amongst them the Stx receptor Gb3 [50].

We recently showed that a 24 h preincubation with 20 µM HG strongly protected HEp-2, HMEC-1 and HBMEC cells against Shiga toxin and Stx2, with an average 30-fold increase of IC50 [50]. There was a moderate reduction in Stx1 binding to HEp-2 cells after HG treatment, presumably due to reduced Gb3 levels, but this decrease was not sufficiently large to account for the strong protection against the toxin. Immunofluorescence confocal microscopy revealed that HG treatment led to an accumulation of Stx1 in the Golgi apparatus after 4 h of toxin challenge, while in control cells, the toxin clearly colocalized with both the ER-marker PDI and the Golgi-marker TGN46 at the same time point, suggesting that HG treatment inhibits Golgi-to-ER transport of Stx [50]. Thus, HG prevents Stx intoxication primarily by interfering with the Golgi-to-ER transport of Stx, but also to some degree by downregulating the Stx receptor Gb3.

The 1-*O*-alkylglycerols, including HG, are naturally occurring ether lipids that are present in human cells and body fluids, particularly in hematopoietic organs and in neutrophils and human milk [169]. Alkylglycerols can also be obtained from the diet, with marine oils, especially shark liver oil, being a good source [169,170]. Shark liver oil contains 9%–13% HG and has been used in traditional medicine in the Nordic countries for beneficial health effects and for wound healing [169,170]. Shark liver oil and alkylglycerol supplementation have been reported to mediate several biological effects, including the ability to boost the immune system and alleviate radiation therapy-induced side effects [169,170]. However, to evaluate the potential of HG as an inhibitor of Stx intoxication, further studies, first in animal models, are required. 

### 2.7. Statins

Statins, also known as HMG-CoA (3-hydroxy-3-methylglutaryl coenzyme A) reductase inhibitors, are a class of compounds that inhibit cholesterol biosynthesis and prenylation of proteins (reviewed in [171]). Statins are widely prescribed to lower serum cholesterol levels for the prevention of cardiac diseases [172,173,174]. Recently, statins were found to inhibit Stx1B transport to the Golgi apparatus and to protect cells against Stx1 and Stx2 [110]. ACHN (epithelial carcinoma from renal tubular adenocarcinoma pleural metastasis) cells were pretreated with rosuvastatin (5–20 µM) for 24 h prior to addition of Stx1 or Stx2, and the number of surviving cells was measured after 72 h. Treatment with rosuvastatin increased cell survival by approx. 25% in the group treated with 5 µM rosuvastatin and then challenged with 1 pg/mL of Stx1 or Stx2, but there was essentially no improvement in cell survival in the group challenged with 100 pg/mL of the toxins. On the other hand, cell treatment with 10 µM of rosuvastatin increased cell survival by approx 70% in the groups challenged with 1 pg/mL of Stx1 or Stx2, and by approx. 25% in the groups challenged with100 pg/mL of the toxins. Although increasing the concentrations of rosuvastatin to 20 µM showed even better protection, there seemed to appear a difference in protection against Stx1 and Stx2, with the protection against Stx2 being lower than that against Stx1, especially at high toxin doses. As previously mentioned, Stx2 is suggested to exploit an additional transport pathway [65] which might not be blocked by rosuvastatin. In addition, a protective effect by lovastatin has previously been shown for several other protein toxins, including ricin, modeccin, *Pseudomonas* toxin, and diphtheria toxin, suggesting that statins might have a potential to be used against several toxins [175]. 

Cell treatment with statins was found to increase the protein level and activity of GlcCer synthase, which in turn led to increased cellular levels of GlcCer [110]. The fact that the cells were maintained in a medium containing cholesterol during the treatment with statins, and that the isoprenol precursor counteracted the upregulation of GlcCer synthesis induced by statins, indicated that the effects observed, at least for the upregulation of GlcCer synthesis, were mediated by the inhibition of isoprenylation rather than by depletion of cellular cholesterol. In addition, by the use of specific inhibitors of different prenylation enzymes, the authors were able to show that it was only the inhibition of geranylgeranyl transferase II, also known as Rab geranylgeranyl transferase, that led to similar upregulation of GlcCer as statin treatment. Rab prenylation facilitates their membrane association and activity [176,177]. Rab GTPases regulate intracellular vesicular trafficking events, and several Rabs have also been implicated in Stx retrograde transport (reviewed in [1]), suggesting that rosuvastatin-induced protection against Stx is mediated via aberrant Rab prenylation. 

Importantly, although for the prevention of cholesterol-related cardiac diseases statins are prescribed in relatively low doses (recommended daily dose of lovastatin is 20–40 mg per day [174]) the achievable safe serum values might be as high as 0.2–12 µM (shown for lovastatin at doses of 133–412 mg/m^2^, which corresponds to approx. 200–650 mg) [178], and are within the dose range found to protect cells against Stx1 and Stx2 [110]. 

### 2.8. Furin Inhibitors

Furin is a type I transmembrane serine protease that activates precursors of different physiologically important proteins. Although furin is mainly located in the Golgi and *trans*-Golgi network, it also circulates through the endosomal system to the cell surface and back to the Golgi [179], and may also be secreted as a soluble truncated active enzyme [180]. In addition to its physiological role, furin activates numerous toxic proteins, including Stxs [95]. Moreover, furin has been implicated in various human diseases, including cancer, osteoarthritis, atherosclerosis, diabetes and neurodegenerative disorders [181,182,183,184]. Although the complete knockout of the *fur* gene, which encodes furin, is embryonically lethal to mice, the specific inhibition of furin by polyarginine inhibitors has been shown to be well tolerated in adult animals [185,186], indicating that short term inhibition of furin might be exploited for treatment. Thus, various furin inhibitors have been developed and investigated for their potential therapeutic applications during the last years [111,112,183,184,187]. For instance, the furin inhibitor hexa-d-arginine amide has been demonstrated to improve the survival of mice challenged with *Pseudomonas aeruginosa* exotoxin A, which requires furin-mediated cleavage for toxicity [185]. Later, the same compound was shown to delay anthrax toxin-induced toxemia both in cells and in rats [186], supporting the therapeutic potential of furin inhibitors for the treatment of infectious diseases. 

A number of peptidomimetic furin inhibitors have been developed and optimized to improve the activity and stability of the compounds [111], and some of these compounds have been found to protect against Shiga toxin without showing significant toxicity to cells [111]. The most effective analogue for the protection against Shiga toxin was 4-(guanidinomethyl)phenylacetyl-Arg-Val-Arg-4-amidinobenzylamide (called No. 24 in the original article), which reduced HEp-2 cells sensitivity to Shiga toxin by approx. 6-fold, when added to the cells 30 min before a 4 h incubation with unnicked Shiga toxin [111]. However, it has not yet been investigated whether cells are protected against longer challenges with the toxin. It has been shown that the Shiga toxin may be processed by other cellular proteases, but more slowly [39,95]. Thus, it is still not clear whether furin inhibitors help to prevent or treat HUS in humans infected with Stx-producing bacteria.

## 3. Concluding Remarks

A number of compounds that protect cells against Stxs are known, and in some cases they have also been found to protect animals against the challenge with purified toxin or toxic effects of infection with STEC. Most of these compounds have not been used in humans and thus need to be carefully evaluated for potential use in the clinic, and it is not clear to which extent one will be able to find compounds that are safe in humans. There are, however, some exceptions, for instance chloroquine and statins, which should be investigated for a possible protective effect in connection with Stx-induced disease. In addition, there is an ongoing search for efficient inhibitors of glycosphingolipid synthesis for the treatment of glycosphingolipid storage diseases [188] as well as cancer [189], meaning that new, more potentially useful compounds will be investigated in the near future. Such new compounds should not be overlooked for their potential in the treatment of STEC and *Shigella* infections.

## Figures and Tables

**Table 1:** Compounds that protect cells against Stx.

Compound	Cellular Action	Targeted Step of Stx Intoxication	Cell Lines Tested	In Vivo Studies	Reference(s)
CQ	Elevation of pH in acidified organelles	Translocation of A_1_-moiety to cytosol (predicted)	HEp-2	-	[98]
Baf	V-ATPase inhibitor	Transport to the Golgi	HEp-2	-	[98]
ConA	V-ATPase inhibitor	Transport to the Golgi	HEp-2	-	[98]
Nig	Ionophore that exchanges H^+^ for monovalent cations	Not determined; later than Golgi	HEp-2	-	[98]
2DG	Inhibition of glycolysis and protein *N*-glycosylation; Ca^2+^ release from the endoplasmic reticulum (ER); Inhibition of Gb3 synthesis	Release of A_1_-moiety	HEp-2, HT-29, SW480, HeLa	-	[99]
FDG	Inhibition of glycolysis and protein *N*-glycosylation; Ca^2+^ release from the ER; Inhibition of GlcCer synthesis	Binding; Transport from Golgi to ER; Release of A_1_-moiety	HEp-2, HT-29, MCF-7, HBMEC	-	[48]
Retro-2 substances	Relocalization of Syntaxins 5 and 6	Transport from endosomes to the Golgi	A459, HeLa	Reduction in mortality rate from 70% to 40% in mice infected with *E. coli* O104:H4	[100,101]
Mn^2+^	Induction of GPP130 oligomerization and its sorting to lysosomes for degradation	Transport from endosomes to the Golgi (no effect on Stx2 transport)	HeLa, Vero	Protection against lethal doses of Stx1 in BALB/c mice; No protection against either Stx1 or Stx2 in CD-1 mice	[65,102,103,104,105]
PDMP	Inhibition of GlcCer synthesis	Binding and endocytosis; Transport from endosomes to the Golgi	HEp-2	-	[43]
C-9	Inhibition of GlcCer synthesis	Not investigated	Human renal tubular epithelial cells, Human glomerular endothelial cells	50% reduction in mortality rate in rats injected with supernatant from *E. coli* expressing Stx2	[106,107,108]
HG	Ether lipid precursor	Transport from Golgi to ER	HEp-2, HMEC-1, HBMEC	-	[109]
Rosuvastatin	Inhibition of cholesterol biosynthesis and protein prenylation	Transport to the Golgi	ACHN	-	[110]
Furin inhibitors	Inhibition of furin	Proteolytic cleavage of the A-moiety	HEp-2	-	[111,112]

## Abbreviations

ACHN	epithelial carcinoma from renal tubular adenocarcinoma pleural metastasis
Baf	bafilomycin A1
ConA	concanamycin A
cPLA2	cytoplasmic phospholipase A2
CQ	chloroquine
C-9	(1R, 2R)-nonanoic acid [2-(2′,3′-dihydro-benzo [1–4]dioxin-6′-yl)-2-hydroxy-1-pyrrolidin-1-ylmethyl-ethyl]-amide-L-tartaric acid salt
EC50	the concentration of the drug that gives 50% of its full inhibitory effect against the toxin
FDG	2-fluoro-2-deoxy-d-glucose
Gal	galactose
Gb3	globotriaosylceramide
Glc	glucose
GlcCer	glucosylceramide
GPP130	Golgi phosphoprotein of 130 kDa
HBMEC	human brain microvascular endothelial cells
HCQ	hydroxychloroquine
HG	alkylglycerol 1-*O*-hexadecyl-*sn*-glycerol
HRTEC	human renal tubular epithelial cells
HUS	hemolytic-uremic syndrome
HuSAP	human serum amyloid component P
IC50	Stx concentration needed to inhibit protein synthesis by 50%
LacCer	lactosylceramide
LPI	lysophosphatidylinositol
Nig	nigericin
PDMP	1-phenyl-2-decanoyl-amino-3-morpholino-1- propanol
PET	positron emission tomography
Retro-2^cycl^	cyclic form of Retro-2
STEC	Shiga toxin-producing *E. coli*
Stx	Shiga toxins (when referring to the whole family and common features)
StxB	B-moiety of Stx
Stx1	Shiga-like toxin 1
Stx2	Shiga-like toxin 2
TLC	thin layer chromatography
UDP	uridine diphosphate
[^18^F]FDG	FDG which contains the ^18^F radioisotope
2DG	2-deoxy-d-glucose

## References

[1] Bergan J., Dyve Lingelem A.B., Simm R., Skotland T., Sandvig K. (2012). Shiga toxins. Toxicon.

[2] Scheutz F., Teel L.D., Beutin L., Piérard D., Buvens G., Karch H., Mellmann A., Caprioli A., Tozzoli R., Morabito S. (2012). Multicenter evaluation of a sequence-based protocol for subtyping Shiga toxins and standardizing Stx nomenclature. J. Clin. Microbiol..

[3] Tesh V.L., Burris J.A., Owens J.W., Gordon V.M., Wadolkowski E.A., O’Brien A.D., Samuel J.E. (1993). Comparison of the relative toxicities of Shiga-like toxins type I and type II for mice. Infect. Immun..

[4] Siegler R.L., Obrig T.G., Pysher T.J., Tesh V.L., Denkers N.D., Taylor F.B. (2003). Response to Shiga toxin 1 and 2 in a baboon model of hemolytic uremic syndrome. Pediatr. Nephrol..

[5] Karch H., Tarr P.I., Bielaszewska M. (2005). Enterohaemorrhagic *Escherichia coli* in human medicine. Int. J. Med. Microbiol..

[6] Palermo M.S., Exeni R.A., Fernandez G.C. (2009). Hemolytic uremic syndrome: pathogenesis and update of interventions. Expert Rev. Anti Infect. Ther..

[7] Agger M., Scheutz F., Villumsen S., Molbak K., Petersen A.M. (2015). Antibiotic treatment of verocytotoxin-producing *Escherichia coli* (VTEC) infection: a systematic review and a proposal. J. Antimicrob. Chemother..

[8] Barton Behravesh C., Jones T.F., Vugia D.J., Long C., Marcus R., Smith K., Thomas S., Zansky S., Fullerton K.E., Henao O.L. (2011). Deaths associated with bacterial pathogens transmitted commonly through food: foodborne diseases active surveillance network (FoodNet), 1996–2005. J. Infect. Dis..

[9] Boisen N., Melton-Celsa A.R., Scheutz F., O’Brien A.D., Nataro J.P. (2015). Shiga toxin 2a and Enteroaggregative *Escherichia coli*—a deadly combination. Gut Microbes.

[10] Tarr P.I., Gordon C.A., Chandler W.L. (2005). Shiga-toxin-producing *Escherichia coli* and haemolytic uraemic syndrome. Lancet.

[11] Gould L.H., Demma L., Jones T.F., Hurd S., Vugia D.J., Smith K., Shiferaw B., Segler S., Palmer A., Zansky S. (2009). Hemolytic uremic syndrome and death in persons with *Escherichia coli* O157:H7 infection, foodborne diseases active surveillance network sites, 2000–2006. Clin. Infect. Dis..

[12] Brandt J.R., Fouser L.S., Watkins S.L., Zelikovic I., Tarr P.I., Nazar-Stewart V., Avner E.D. (1994). *Escherichia coli* O157:H7-associated hemolytic-uremic syndrome after ingestion of contaminated hamburgers. J. Pediatr..

[13] Hughes D.A., Beattie T.J., Murphy A.V. (1991). Haemolytic uraemic syndrome: 17 years’ experience in a Scottish paediatric renal unit. Scott. Med. J..

[14] Taylor C.M., White R.H., Winterborn M.H., Rowe B. (1986). Haemolytic-uraemic syndrome: Clinical experience of an outbreak in the West Midlands. Br. Med. J. (Clin. Res. Ed.).

[15] Bale J.F., Brasher C., Siegler R.L. (1980). CNS manifestations of the hemolytic-uremic syndrome. Relationship to metabolic alterations and prognosis. Am. J. Dis. Child..

[16] Obata F., Tohyama K., Bonev A.D., Kolling G.L., Keepers T.R., Gross L.K., Nelson M.T., Sato S., Obrig T.G. (2008). Shiga toxin 2 affects the central nervous system through receptor globotriaosylceramide localized to neurons. J. Infect. Dis..

[17] Armstrong G.D., Fodor E., Vanmaele R. (1991). Investigation of Shiga-like toxin binding to chemically synthesized oligosaccharide sequences. J. Infect. Dis..

[18] Trachtman H., Cnaan A., Christen E., Gibbs K., Zhao S., Acheson D.W., Weiss R., Kaskel F.J., Spitzer A., Hirschman G.H. (2003). Effect of an oral Shiga toxin-binding agent on diarrhea-associated hemolytic uremic syndrome in children: a randomized controlled trial. JAMA.

[19] Nishikawa K., Matsuoka K., Kita E., Okabe N., Mizuguchi M., Hino K., Miyazawa S., Yamasaki C., Aoki J., Takashima S. (2002). A therapeutic agent with oriented carbohydrates for treatment of infections by Shiga toxin-producing *Escherichia coli* O157:H7. Proc. Natl. Acad. Sci. USA.

[20] Mulvey G.L., Marcato P., Kitov P.I., Sadowska J., Bundle D.R., Armstrong G.D. (2003). Assessment in mice of the therapeutic potential of tailored, multivalent Shiga toxin carbohydrate ligands. J. Infect. Dis..

[21] Melton-Celsa A.R., Carvalho H.M., Thuning-Roberson C., O’Brien A.D. (2015). Protective efficacy and pharmacokinetics of human/mouse chimeric anti-Stx1 and anti-Stx2 antibodies in mice. Clin. Vaccine Immunol..

[22] Kimura T., Tani S., Matsumoto Yi Y., Takeda T. (2001). Serum amyloid P component is the Shiga toxin 2-neutralizing factor in human blood. J. Biol. Chem..

[23] Armstrong G.D., Mulvey G.L., Marcato P., Griener T.P., Kahan M.C., Tennent G.A., Sabin C.A., Chart H., Pepys M.B. (2006). Human serum amyloid P component protects against *Escherichia coli* O157:H7 Shiga toxin 2 in vivo: Therapeutic implications for hemolytic-uremic syndrome. J. Infect. Dis..

[24] Kielstein J.T., Beutel G., Fleig S., Steinhoff J., Meyer T.N., Hafer C., Kuhlmann U., Bramstedt J., Panzer U., Vischedyk M. (2012). Best supportive care and therapeutic plasma exchange with or without eculizumab in Shiga-toxin-producing *E. coli* O104:H4 induced haemolytic-uraemic syndrome: an analysis of the German STEC-HUS registry. Nephrol. Dial. Transplant..

[25] Orth D., Khan A.B., Naim A., Grif K., Brockmeyer J., Karch H., Joannidis M., Clark S.J., Day A.J., Fidanzi S. (2009). Shiga toxin activates complement and binds factor H: evidence for an active role of complement in hemolytic uremic syndrome. J. Immunol..

[26] Melton-Celsa A.R., O’Brien A.D. (2014). New therapeutic developments against Shiga toxin-producing *Escherichia coli*. Microbiol. Spectr..

[27] Stein P.E., Boodhoo A., Tyrrell G.J., Brunton J.L., Read R.J. (1992). Crystal structure of the cell-binding B oligomer of verotoxin-1 from *E. coli*. Nature.

[28] Fraser M.E., Chernaia M.M., Kozlov Y.V., James M.N. (1994). Crystal structure of the holotoxin from *Shigella dysenteriae* at 2.5 Å resolution. Nat. Struct. Biol..

[29] Jacewicz M., Clausen H., Nudelman E., Donohue-Rolfe A., Keusch G.T. (1986). Pathogenesis of shigella diarrhea. XI. Isolation of a shigella toxin-binding glycolipid from rabbit jejunum and HeLa cells and its identification as globotriaosylceramide. J. Exp. Med..

[30] Lindberg A.A., Brown J.E., Stromberg N., Westling-Ryd M., Schultz J.E., Karlsson K.A. (1987). Identification of the carbohydrate receptor for Shiga toxin produced by *Shigella dysenteriae* type 1. J. Biol. Chem..

[31] Lingwood C.A., Law H., Richardson S., Petric M., Brunton J.L., De G.S., Karmali M. (1987). Glycolipid binding of purified and recombinant *Escherichia coli* produced verotoxin *in vitro*. J. Biol. Chem..

[32] DeGrandis S., Law H., Brunton J., Gyles C., Lingwood C.A. (1989). Globotetraosylceramide is recognized by the pig edema disease toxin. J. Biol. Chem..

[33] Ling H., Boodhoo A., Hazes B., Cummings M.D., Armstrong G.D., Brunton J.L., Read R.J. (1998). Structure of the Shiga-like toxin I B-pentamer complexed with an analogue of its receptor Gb3. Biochemistry.

[34] Peter M.G., Lingwood C.A. (2000). Apparent cooperativity in multivalent verotoxin-globotriaosyl ceramide binding: kinetic and saturation binding studies with [^125^I]verotoxin. Biochim. Biophys Acta.

[35] Bast D.J., Banerjee L., Clark C., Read R.J., Brunton J.L. (1999). The identification of three biologically relevant globotriaosyl ceramide receptor binding sites on the Verotoxin 1 B subunit. Mol. Microbiol..

[36] Marcato P., Mulvey G., Armstrong G.D. (2002). Cloned Shiga toxin 2 B subunit induces apoptosis in Ramos Burkitt’s lymphoma B cells. Infect. Immun..

[37] Kymre L., Simm R., Skotland T., Sandvig K. (2015). Different roles of the C-terminal end of Stx1A and Stx2A for AB_5_ complex integrity and retrograde transport of Stx in HeLa cells. Pathog. Dis..

[38] Fraser M.E., Fujinaga M., Cherney M.M., Melton-Celsa A.R., Twiddy E.M., O’Brien A.D., James M.N. (2004). Structure of Shiga toxin type 2 (Stx2) from *Escherichia coli* O157:H7. J. Biol. Chem..

[39] Garred O., van Deurs B., Sandvig K. (1995). Furin-induced cleavage and activation of Shiga toxin. J. Biol. Chem..

[40] Tam P.J., Lingwood C.A. (2007). Membrane cytosolic translocation of verotoxin A_1_ subunit in target cells. Microbiology.

[41] Zumbrun S.D., Hanson L., Sinclair J.F., Freedy J., Melton-Celsa A.R., Rodriguez-Canales J., Hanson J.C., O’Brien A.D. (2010). Human intestinal tissue and cultured colonic cells contain globotriaosylceramide synthase mRNA and the alternate Shiga toxin receptor globotetraosylceramide. Infect. Immun..

[42] Lingwood C.A., Binnington B., Manis A., Branch D.R. (2010). Globotriaosyl ceramide receptor function—where membrane structure and pathology intersect. FEBS Lett..

[43] Raa H., Grimmer S., Schwudke D., Bergan J., Walchli S., Skotland T., Shevchenko A., Sandvig K. (2009). Glycosphingolipid requirements for endosome-to-Golgi transport of Shiga toxin. Traffic.

[44] Kiarash A., Boyd B., Lingwood C.A. (1994). Glycosphingolipid receptor function is modified by fatty acid content. Verotoxin 1 and verotoxin 2c preferentially recognize different globotriaosyl ceramide fatty acid homologues. J. Biol. Chem..

[45] Pellizzari A., Pang H., Lingwood C.A. (1992). Binding of verocytotoxin 1 to its receptor is influenced by differences in receptor fatty acid content. Biochemistry.

[46] Sandvig K., Bergan J., Kavaliauskiene S., Skotland T. (2014). Lipid requirements for entry of protein toxins into cells. Prog. Lipid Res..

[47] Kavaliauskiene S., Nymark C.-M., Bergan J., Simm R., Sylvanne T., Simolin H., Ekroos K., Skotland T., Sandvig K. (2013). Cell density-induced changes in lipid composition and intracellular trafficking. Cell. Mol. Life Sci..

[48] Kavaliauskiene S., Torgersen M.L., Dyve Lingelem A.B., Klokk T.I., Lintonen T., Simolin H., Ekroos K., Skotland T., Sandvig K. (2016). Cellular effects of fluorodeoxyglucose: Global changes in the lipidome and alteration in intracellular transport. Oncotarget.

[49] Sandvig K., Ryd M., Garred O., Schweda E., Holm P.K., van Deurs B. (1994). Retrograde transport from the Golgi complex to the ER of both Shiga toxin and the nontoxic Shiga B-fragment is regulated by butyric acid and cAMP. J. Cell Biol..

[50] Bergan J., Skotland T., Sylvänne T., Simolin H., Ekroos K., Sandvig K. (2013). The ether lipid precursor hexadecylglycerol causes major changes in the lipidome of HEp-2 cells. PLoS ONE.

[51] Hughes A.K., Stricklett P.K., Schmid D., Kohan D.E. (2000). Cytotoxic effect of Shiga toxin-1 on human glomerular epithelial cells. Kidney Int..

[52] Ramegowda B., Samuel J.E., Tesh V.L. (1999). Interaction of Shiga toxins with human brain microvascular endothelial cells: Cytokines as sensitizing agents. J. Infect. Dis..

[53] Zumbrun S.D., Melton-Celsa A.R., O’Brien A.D. (2014). When a healthy diet turns deadly. Gut Microbes.

[54] Skotland T., Ekroos K., Kavaliauskiene S., Bergan J., Kauhanen D., Lintonen T., Sandvig K. (2016). Determining the turnover of glycosphingolipid species by stable-isotope tracer lipidomics. J. Mol. Biol..

[55] Mahfoud R., Manis A., Lingwood C.A. (2009). Fatty acid-dependent globotriaosyl ceramide receptor function in detergent resistant model membranes. J. Lipid Res..

[56] Lingwood D., Binnington B., Rog T., Vattulainen I., Grzybek M., Coskun U., Lingwood C.A., Simons K. (2011). Cholesterol modulates glycolipid conformation and receptor activity. Nat. Chem. Biol..

[57] Mahfoud R., Manis A., Binnington B., Ackerley C., Lingwood C.A. (2010). A major fraction of glycosphingolipids in model and cellular cholesterol-containing membranes is undetectable by their binding proteins. J. Biol. Chem..

[58] Boerlin P., McEwen S.A., Boerlin-Petzold F., Wilson J.B., Johnson R.P., Gyles C.L. (1999). Associations between virulence factors of Shiga toxin-producing *Escherichia coli* and disease in humans. J. Clin. Microbiol..

[59] Itoh K., Tezuka T., Inoue K., Tada H., Suzuki T. (2001). Different binding property of verotoxin-1 and verotoxin-2 against their glycolipid receptor, globotriaosylceramide. Tohoku J. Exp. Med..

[60] Head S.C., Karmali M.A., Lingwood C.A. (1991). Preparation of VT1 and VT2 hybrid toxins from their purified dissociated subunits. Evidence for B subunit modulation of a subunit function. J. Biol. Chem..

[61] Rutjes N.W., Binnington B.A., Smith C.R., Maloney M.D., Lingwood C.A. (2002). Differential tissue targeting and pathogenesis of verotoxins 1 and 2 in the mouse animal model. Kidney Int..

[62] Chark D., Nutikka A., Trusevych N., Kuzmina J., Lingwood C. (2004). Differential carbohydrate epitope recognition of globotriaosyl ceramide by verotoxins and a monoclonal antibody. Eur. J. Biochem..

[63] Khan F., Proulx F., Lingwood C.A. (2009). Detergent-resistant globotriaosyl ceramide may define verotoxin/glomeruli-restricted hemolytic uremic syndrome pathology. Kidney Int..

[64] Tam P., Mahfoud R., Nutikka A., Khine A.A., Binnington B., Paroutis P., Lingwood C. (2008). Differential intracellular transport and binding of verotoxin 1 and verotoxin 2 to globotriaosylceramide-containing lipid assemblies. J. Cell. Physiol..

[65] Mukhopadhyay S., Redler B., Linstedt A.D. (2013). Shiga toxin-binding site for host cell receptor GPP130 reveals unexpected divergence in toxin-trafficking mechanisms. Mol. Biol. Cell.

[66] Selyunin A.S., Mukhopadhyay S. (2015). A conserved structural motif mediates retrograde trafficking of Shiga toxin types 1 and 2. Traffic.

[67] Ergonul Z., Clayton F., Fogo A.B., Kohan D.E. (2003). Shigatoxin-1 binding and receptor expression in human kidneys do not change with age. Pediatr. Nephrol..

[68] Lingwood C.A. (1994). Verotoxin-binding in human renal sections. Nephron.

[69] Boyd B., Lingwood C. (1989). Verotoxin receptor glycolipid in human renal tissue. Nephron.

[70] Ohmi K., Kiyokawa N., Takeda T., Fujimoto J. (1998). Human microvascular endothelial cells are strongly sensitive to Shiga toxins. Biochem. Biophys. Res. Commun..

[71] Miyamoto Y., Iimura M., Kaper J.B., Torres A.G., Kagnoff M.F. (2006). Role of Shiga toxin versus H7 flagellin in enterohaemorrhagic *Escherichia coli* signalling of human colon epithelium *in vivo*. Cell. Microbiol..

[72] Ren J., Utsunomiya I., Taguchi K., Ariga T., Tai T., Ihara Y., Miyatake T. (1999). Localization of verotoxin receptors in nervous system. Brain Res..

[73] Cooling L.L., Walker K.E., Gille T., Koerner T.A. (1998). Shiga toxin binds human platelets via globotriaosylceramide (P^k^ antigen) and a novel platelet glycosphingolipid. Infect. Immun..

[74] Tao R.V., Sweeley C.C., Jamieson G.A. (1973). Sphingolipid composition of human platelets. J. Lipid Res..

[75] Steffensen R., Carlier K., Wiels J., Levery S.B., Stroud M., Cedergren B., Nilsson S.B., Bennett E.P., Jersild C., Clausen H. (2000). Cloning and expression of the histo-blood group P^k^ UDP-galactose: Galβ1-4G1cβ1-Cer α1,4-galactosyltransferase. Molecular genetic basis of the p phenotype. J. Biol. Chem..

[76] Mangeney M., Richard Y., Coulaud D., Tursz T., Wiels J. (1991). CD77: An antigen of germinal center B cells entering apoptosis. Eur. J. Immunol..

[77] Engedal N., Skotland T., Torgersen M.L., Sandvig K. (2011). Shiga toxin and its use in targeted cancer therapy and imaging. Microb. Biotechnol..

[78] Okuda T., Tokuda N., Numata S., Ito M., Ohta M., Kawamura K., Wiels J., Urano T., Tajima O., Furukawa K. (2006). Targeted disruption of Gb3/CD77 synthase gene resulted in the complete deletion of globo-series glycosphingolipids and loss of sensitivity to verotoxins. J. Biol. Chem..

[79] Lauvrak S.U., Walchli S., Iversen T.G., Slagsvold H.H., Torgersen M.L., Spilsberg B., Sandvig K. (2006). Shiga toxin regulates its entry in a Syk-dependent manner. Mol. Biol. Cell.

[80] Katagiri Y.U., Mori T., Nakajima H., Katagiri C., Taguchi T., Takeda T., Kiyokawa N., Fujimoto J. (1999). Activation of Src family kinase yes induced by Shiga toxin binding to globotriaosyl ceramide (Gb3/CD77) in low density, detergent-insoluble microdomains. J. Biol. Chem..

[81] Mori T., Kiyokawa N., Katagiri Y.U., Taguchi T., Suzuki T., Sekino T., Sato N., Ohmi K., Nakajima H., Takeda T. (2000). Globotriaosyl ceramide (CD77/Gb3) in the glycolipid-enriched membrane domain participates in B-cell receptor-mediated apoptosis by regulating lyn kinase activity in human B cells. Exp. Hematol..

[82] Torgersen M.L., Walchli S., Grimmer S., Skanland S.S., Sandvig K. (2007). Protein kinase Cδ is activated by Shiga toxin and regulates its transport. J. Biol. Chem..

[83] Walchli S., Skanland S.S., Gregers T.F., Lauvrak S.U., Torgersen M.L., Ying M., Kuroda S., Maturana A., Sandvig K. (2008). The mitogen-activated protein kinase p38 links Shiga toxin-dependent signaling and trafficking. Mol. Biol. Cell.

[84] Klokk T.I., Kavaliauskiene S., Sandvig K. (2016). Cross-linking of glycosphingolipids at the plasma membrane: consequences for intracellular signaling and traffic. Cell. Mol. Life Sci..

[85] Tcatchoff L., Andersson S., Utskarpen A., Klokk T.I., Skanland S.S., Pust S., Gerke V., Sandvig K. (2012). Annexin A1 and A2: Roles in retrograde trafficking of Shiga toxin. PLoS ONE.

[86] Takenouchi H., Kiyokawa N., Taguchi T., Matsui J., Katagiri Y.U., Okita H., Okuda K., Fujimoto J. (2004). Shiga toxin binding to globotriaosyl ceramide induces intracellular signals that mediate cytoskeleton remodeling in human renal carcinoma-derived cells. J. Cell Sci..

[87] Hehnly H., Longhini K.M., Chen J.L., Stamnes M. (2009). Retrograde Shiga toxin trafficking is regulated by ARHGAP21 and Cdc42. Mol. Biol. Cell.

[88] Hehnly H., Sheff D., Stamnes M. (2006). Shiga toxin facilitates its retrograde transport by modifying microtubule dynamics. Mol. Biol. Cell.

[89] Mallard F., Antony C., Tenza D., Salamero J., Goud B., Johannes L. (1998). Direct pathway from early/recycling endosomes to the Golgi apparatus revealed through the study of Shiga toxin B-fragment transport. J. Cell Biol..

[90] Sandvig K., Olsnes S., Brown J.E., Petersen O.W., van Deurs B. (1989). Endocytosis from coated pits of Shiga toxin: a glycolipid-binding protein from *Shigella dysenteriae* 1. J. Cell Biol..

[91] Sandvig K., Garred O., Prydz K., Kozlov J.V., Hansen S.H., van Deurs B. (1992). Retrograde transport of endocytosed Shiga toxin to the endoplasmic reticulum. Nature.

[92] Donta S.T., Tomicic T.K., Donohue-Rolfe A. (1995). Inhibition of Shiga-like toxins by brefeldin A. J. Infect. Dis..

[93] Sandvig K., Prydz K., Ryd M., van Deurs B. (1991). Endocytosis and intracellular transport of the glycolipid-binding ligand Shiga toxin in polarized MDCK cells. J. Cell Biol..

[94] Garred O., Dubinina E., Polesskaya A., Olsnes S., Kozlov J., Sandvig K. (1997). Role of the disulfide bond in Shiga toxin A-chain for toxin entry into cells. J. Biol. Chem..

[95] Garred O., Dubinina E., Holm P.K., Olsnes S., van Deurs B., Kozlov J.V., Sandvig K. (1995). Role of processing and intracellular transport for optimal toxicity of Shiga toxin and toxin mutants. Exp. Cell Res..

[96] Johannes L., Romer W. (2010). Shiga toxin—From cell biology to biomedical applications. Nat. Rev. Microbiol..

[97] Lee M.S., Koo S., Jeong D.G., Tesh V.L. (2016). Shiga toxins as multi-functional proteins: Induction of host cellular stress responses, role in pathogenesis and therapeutic applications. Toxins (Basel).

[98] Dyve Lingelem A.B., Bergan J., Sandvig K. (2012). Inhibitors of intravesicular acidification protect against Shiga toxin in a pH-independent manner. Traffic.

[99] Kavaliauskiene S., Skotland T., Sylvanne T., Simolin H., Klokk T.I., Torgersen M.L., Lingelem A.B., Simm R., Ekroos K., Sandvig K. (2015). Novel actions of 2-deoxy-d-glucose: protection against Shiga toxins and changes in cellular lipids. Biochem. J..

[100] Stechmann B., Bai S.K., Gobbo E., Lopez R., Merer G., Pinchard S., Panigai L., Tenza D., Raposo G., Beaumelle B. (2010). Inhibition of retrograde transport protects mice from lethal ricin challenge. Cell.

[101] Secher T., Shima A., Hinsinger K., Cintrat J.C., Johannes L., Barbier J., Gillet D., Oswald E. (2015). Retrograde trafficking inhibitor of Shiga toxins reduces morbidity and mortality of mice infected with enterohemorrhagic *Esherichia coli*. Antimicrob. Agents Chemother..

[102] Tewari R., Jarvela T., Linstedt A.D. (2014). Manganese induces oligomerization to promote down-regulation of the intracellular trafficking receptor used by Shiga toxin. Mol. Biol. Cell.

[103] Mukhopadhyay S., Linstedt A.D. (2012). Manganese blocks intracellular trafficking of Shiga toxin and protects against Shiga toxicosis. Science.

[104] Gaston M.A., Pellino C.A., Weiss A.A. (2013). Failure of manganese to protect from Shiga toxin. PLoS ONE.

[105] Sandvig K., Brown J.E. (1987). Ionic requirements for entry of Shiga toxin from *Shigella dysenteriae* 1 into cells. Infect. Immun..

[106] Silberstein C., Copeland D.P., Chiang W., Repetto H.A., Ibarra C. (2008). A glucosylceramide synthase inhibitor prevents the cytotoxic effects of Shiga toxin-2 on human renal tubular epithelial cells. J. Epithel. Biol. Pharmacol..

[107] Silberstein C., Lucero M.S., Zotta E., Copeland D.P., Lingyun L., Repetto H.A., Ibarra C. (2011). A glucosylceramide synthase inhibitor protects rats against the cytotoxic effects of Shiga toxin 2. Pediatr. Res..

[108] Amaral M.M., Sacerdoti F., Jancic C., Repetto H.A., Paton A.W., Paton J.C., Ibarra C. (2013). Action of Shiga toxin type-2 and subtilase cytotoxin on human microvascular endothelial cells. PLoS ONE.

[109] Bergan J., Skotland T., Lingelem A.B., Simm R., Spilsberg B., Lindback T., Sylvanne T., Simolin H., Ekroos K., Sandvig K. (2014). The ether lipid precursor hexadecylglycerol protects against Shiga toxins. Cell. Mol. Life Sci..

[110] Binnington B., Nguyen L., Kamani M., Hossain D., Marks D.L., Budani M., Lingwood C.A. (2016). Inhibition of Rab prenylation by statins induces cellular glycosphingolipid remodeling. Glycobiology.

[111] Becker G.L., Lu Y., Hardes K., Strehlow B., Levesque C., Lindberg I., Sandvig K., Bakowsky U., Day R., Garten W. (2012). Highly potent inhibitors of proprotein convertase furin as potential drugs for treatment of infectious diseases. J. Biol. Chem..

[112] Hardes K., Becker G.L., Lu Y., Dahms S.O., Kohler S., Beyer W., Sandvig K., Yamamoto H., Lindberg I., Walz L. (2015). Novel Furin inhibitors with potent anti-infectious activity. ChemMedChem.

[113] Meshnick S.R., Dobson M.J., Rosenthal P.J. (2001). The history of antimalarial drugs. Antimalarial Chemotherapy: Mechanisms of Action, Resistance, and New Directions in Drug Discovery.

[114] Kitchen L.W., Vaughn D.W., Skillman D.R. (2006). Role of US military research programs in the development of US Food and Drug Administration—Approved antimalarial drugs. Clin. Infect. Dis..

[115] Al-Bari M.A. (2015). Chloroquine analogues in drug discovery: New directions of uses, mechanisms of actions and toxic manifestations from malaria to multifarious diseases. J. Antimicrob. Chemother..

[116] Pascolo S. (2016). Time to use a dose of chloroquine as an adjuvant to anti-cancer chemotherapies. Eur. J. Pharmacol..

[117] Keeling D.J., Herslof M., Ryberg B., Sjogren S., Solvell L. (1997). Vacuolar H(+)-ATPases. Targets for drug discovery?. Ann. N. Y. Acad. Sci..

[118] Parra K.J., Tegos G., Mylonakis E. (2012). Vacuolar ATPase: A model proton pump for antifungal drug discovery. Antimicrobial Drug Discovery: Emerging Strategies.

[119] Moreau D., Kumar P., Wang S.C., Chaumet A., Chew S.Y., Chevalley H., Bard F. (2011). Genome-wide RNAi screens identify genes required for Ricin and PE intoxications. Dev. Cell.

[120] Yu M., Haslam D.B. (2005). Shiga toxin is transported from the endoplasmic reticulum following interaction with the luminal chaperone HEDJ/ERDj3. Infect. Immun..

[121] Rodriguez-Menchaca A.A., Navarro-Polanco R.A., Ferrer-Villada T., Rupp J., Sachse F.B., Tristani-Firouzi M., Sanchez-Chapula J.A. (2008). The molecular basis of chloroquine block of the inward rectifier Kir2.1 channel. Proc. Natl. Acad. Sci. USA.

[122] Orlik F., Schiffler B., Benz R. (2005). Anthrax toxin protective antigen: inhibition of channel function by chloroquine and related compounds and study of binding kinetics using the current noise analysis. Biophys J..

[123] Bachmeyer C., Benz R., Barth H., Aktories K., Gilbert M., Popoff M.R. (2001). Interaction of *Clostridium botulinum* C2 toxin with lipid bilayer membranes and Vero cells: inhibition of channel function by chloroquine and related compounds *in vitro* and intoxification *in vivo*. FASEB J..

[124] Browning D.J. (2014). Pharmacology of chloroquine and hydroxychloroquine. Hydroxychloroquine and Chloroquine Retinopathy.

[125] Molina D.K. (2012). Postmortem hydroxychloroquine concentrations in nontoxic cases. Am. J. Forensic Med. Pathol..

[126] Brown J. (1962). Effects of 2-deoxyglucose on carbohydrate metablism: review of the literature and studies in the rat. Metabolism.

[127] Wick A.N., Drury D.R., Nakada H.I., Wolfe J.B. (1957). Localization of the primary metabolic block produced by 2-deoxyglucose. J. Biol. Chem..

[128] Cramer F.B., Woodward G.E. (1952). 2-Desoxy-d-glucose as an antagonist of glucose in yeast fermentation. J. Frankl. Inst..

[129] Sols A., Crane R.K. (1954). Substrate specificity of brain hexokinase. J. Biol. Chem..

[130] Chen W., Gueron M. (1992). The inhibition of bovine heart hexokinase by 2-deoxy-d-glucose-6-phosphate: characterization by ^31^P NMR and metabolic implications. Biochimie.

[131] Datema R., Schwarz R.T. (1979). Interference with glycosylation of glycoproteins. Inhibition of formation of lipid-linked oligosaccharides *in vivo*. Biochem. J..

[132] Desselle A., Chaumette T., Gaugler M.H., Cochonneau D., Fleurence J., Dubois N., Hulin P., Aubry J., Birkle S., Paris F. (2012). Anti-Gb3 monoclonal antibody inhibits angiogenesis and tumor development. PLoS ONE.

[133] Watowich S.S., Morimoto R.I. (1988). Complex regulation of heat shock- and glucose-responsive genes in human cells. Mol. Cell. Biol..

[134] Shinjo S., Mizotani Y., Tashiro E., Imoto M. (2013). Comparative analysis of the expression patterns of UPR-target genes caused by UPR-inducing compounds. Biosci. Biotechnol. Biochem..

[135] Okuda T., Furukawa K., Nakayama K.I. (2009). A novel, promoter-based, target-specific assay identifies 2-deoxy-d-glucose as an inhibitor of globotriaosylceramide biosynthesis. FEBS J..

[136] Kurtoglu M., Maher J.C., Lampidis T.J. (2007). Differential toxic mechanisms of 2-deoxy-d-glucose versus 2-fluorodeoxy-d-glucose in hypoxic and normoxic tumor cells. Antioxid. Redox Signal..

[137] Lampidis T.J., Kurtoglu M., Maher J.C., Liu H., Krishan A., Sheft V., Szymanski S., Fokt I., Rudnicki W.R., Ginalski K. (2006). Efficacy of 2-halogen substituted d-glucose analogs in blocking glycolysis and killing “hypoxic tumor cells”. Cancer Chemother. Pharmacol..

[138] Datema R., Schwarz R.T., Jankowski A.W. (1980). Fluoroglucose-inhibition of protein glycosylation *in vivo*. Inhibition of mannose and glucose incorporation into lipid-linked oligosaccharides. Eur. J. Biochem..

[139] Schmidt M.F., Biely P., Kratky Z., Schwarz R.T. (1978). Metabolism of 2-deoxy-2-fluoro-d-[^3^H]glucose and 2-deoxy-2-fluoro-d-[^3^H]mannose in yeast and chick-embryo cells. Eur. J. Biochem..

[140] Hoh C.K. (2007). Clinical use of FDG-PET. Nucl. Med. Biol..

[141] Kelloff G.J., Hoffman J.M., Johnson B., Scher H.I., Siegel B.A., Cheng E.Y., Cheson B.D., O’Shaughnessy J., Guyton K.Z., Mankoff D.A. (2005). Progress and promise of FDG-PET imaging for cancer patient management and oncologic drug development. Clin. Cancer. Res..

[142] Xi H., Barredo J.C., Merchan J.R., Lampidis T.J. (2013). Endoplasmic reticulum stress induced by 2-deoxyglucose but not glucose starvation activates AMPK through CAMKKβ leading to autophagy. Biochem. Pharmacol..

[143] Xi H., Kurtoglu M., Liu H., Wangpaichitr M., You M., Liu X., Savaraj N., Lampidis T.J. (2011). 2-Deoxy-d-glucose activates autophagy via endoplasmic reticulum stress rather than ATP depletion. Cancer Chemother. Pharmacol..

[144] Biswas C., Ostrovsky O., Makarewich C.A., Wanderling S., Gidalevitz T., Argon Y. (2007). The peptide-binding activity of GRP94 is regulated by calcium. Biochem. J..

[145] Hendershot L.M. (2004). The ER function BiP is a master regulator of ER function. Mt. Sinai J. Med..

[146] Raez L.E., Papadopoulos K., Ricart A.D., Chiorean E.G., Dipaola R.S., Stein M.N., Rocha Lima C.M., Schlesselman J.J., Tolba K., Langmuir V.K. (2013). A phase I dose-escalation trial of 2-deoxy-d-glucose alone or combined with docetaxel in patients with advanced solid tumors. Cancer Chemother. Pharmacol..

[147] Saenz J.B., Doggett T.A., Haslam D.B. (2007). Identification and characterization of small molecules that inhibit intracellular toxin transport. Infect. Immun..

[148] Park J.G., Kahn J.N., Tumer N.E., Pang Y.P. (2012). Chemical structure of Retro-2, a compound that protects cells against ribosome-inactivating proteins. Sci. Rep..

[149] Noel R., Gupta N., Pons V., Goudet A., Garcia-Castillo M.D., Michau A., Martinez J., Buisson D.A., Johannes L., Gillet D. (2013). *N*-methyldihydroquinazolinone derivatives of Retro-2 with enhanced efficacy against Shiga toxin. J. Med. Chem..

[150] Carney D.W., Nelson C.D., Ferris B.D., Stevens J.P., Lipovsky A., Kazakov T., DiMaio D., Atwood W.J., Sello J.K. (2014). Structural optimization of a retrograde trafficking inhibitor that protects cells from infections by human polyoma- and papillomaviruses. Bioorg. Med. Chem..

[151] Gupta N., Pons V., Noel R., Buisson D.A., Michau A., Johannes L., Gillet D., Barbier J., Cintrat J.C. (2014). (*S*)-*N*-Methyldihydroquinazolinones are the active enantiomers of Retro-2 derived compounds against toxins. ACS Med. Chem. Lett..

[152] Frank C., Werber D., Cramer J.P., Askar M., Faber M., an der Heiden M., Bernard H., Fruth A., Prager R., Spode A. (2011). Epidemic profile of Shiga-toxin-producing *Escherichia coli* O104:H4 outbreak in Germany. N. Engl. J. Med..

[153] Bielaszewska M., Mellmann A., Zhang W., Kock R., Fruth A., Bauwens A., Peters G., Karch H. (2011). Characterisation of the *Escherichia coli* strain associated with an outbreak of haemolytic uraemic syndrome in Germany, 2011: a microbiological study. Lancet Infect. Dis..

[154] Gupta N., Noel R., Goudet A., Hinsinger K., Michau A., Pons V., Abdelkafi H., Secher T., Shima A., Shtanko O. (2016). Inhibitors of retrograde trafficking active against ricin and Shiga toxins also protect cells from several viruses, *Leishmania* and Chlamydiales. Chem. Biol. Interact..

[155] Aschner M., Erikson K.M., Herrero Hernandez E., Tjalkens R. (2009). Manganese and its role in Parkinson’s disease: from transport to neuropathology. Neuromol. Med..

[156] Racette B.A., Aschner M., Guilarte T.R., Dydak U., Criswell S.R., Zheng W. (2012). Pathophysiology of manganese-associated neurotoxicity. Neurotoxicology.

[157] Vunnam R.R., Radin N.S. (1980). Analogs of ceramide that inhibit glucocerebroside synthetase in mouse brain. Chem. Phys. Lipids.

[158] Jmoudiak M., Futerman A.H. (2005). Gaucher disease: pathological mechanisms and modern management. Br. J. Haematol..

[159] Barbour S., Edidin M., Felding-Habermann B., Taylor-Norton J., Radin N.S., Fenderson B.A. (1992). Glycolipid depletion using a ceramide analogue (PDMP) alters growth, adhesion, and membrane lipid organization in human A431 cells. J. Cell Physiol..

[160] Arab S., Lingwood C.A. (1998). Intracellular targeting of the endoplasmic reticulum/nuclear envelope by retrograde transport may determine cell hypersensitivity to verotoxin via globotriaosyl ceramide fatty acid isoform traffic. J. Cell. Physiol..

[161] Sandvig K., Garred O., van Helvoort A., van Meer G., van Deurs B. (1996). Importance of glycolipid synthesis for butyric acid-induced sensitization to Shiga toxin and intracellular sorting of toxin in A431 cells. Mol. Biol. Cell.

[162] Lee L., Abe A., Shayman J.A. (1999). Improved inhibitors of glucosylceramide synthase. J. Biol. Chem..

[163] Basu M., Dastgheib S., Girzadas M.A., O’Donnell P.H., Westervelt C.W., Li Z., Inokuchi J., Basu S. (1998). Hydrophobic nature of mammalian ceramide glycanases: purified from rabbit and rat mammary tissues. Acta Biochim. Pol..

[164] Abe A., Gregory S., Lee L., Killen P.D., Brady R.O., Kulkarni A., Shayman J.A. (2000). Reduction of globotriaosylceramide in Fabry disease mice by substrate deprivation. J. Clin. Invest..

[165] Poole R.M. (2014). Eliglustat: first global approval. Drugs.

[166] Belmatoug N., Di Rocco M., Fraga C., Giraldo P., Hughes D., Lukina E., Maison-Blanche P., Merkel M., Niederau C., Plckinger U. (2016). Management and monitoring recommendations for the use of eliglustat in adults with type 1 Gaucher disease in Europe. Eur. J. Intern. Med..

[167] Lukina E., Watman N., Arreguin E.A., Banikazemi M., Dragosky M., Iastrebner M., Rosenbaum H., Phillips M., Pastores G.M., Rosenthal D.I. (2010). A phase 2 study of eliglustat tartrate (Genz-112638), an oral substrate reduction therapy for Gaucher disease type 1. Blood.

[168] Das A.K., Holmes R.D., Wilson G.N., Hajra A.K. (1992). Dietary ether lipid incorporation into tissue plasmalogens of humans and rodents. Lipids.

[169] Iannitti T., Palmieri B. (2010). An update on the therapeutic role of alkylglycerols. Mar. Drugs.

[170] Deniau A.L., Mosset P., Pedrono F., Mitre R., Le Bot D., Legrand A.B. (2010). Multiple beneficial health effects of natural alkylglycerols from shark liver oil. Mar. Drugs.

[171] Matusewicz L., Meissner J., Toporkiewicz M., Sikorski A.F. (2015). The effect of statins on cancer cells—Review. Tumour Biol..

[172] Correale M., Abruzzese S., Greco C.A., Concilio M., Di Biase M., Brunetti N.D. (2012). Statins in heart failure. Curr. Vasc. Pharmacol..

[173] Paliani U., Ricci S. (2012). The role of statins in stroke. Intern. Emerg. Med..

[174] Bibbins-Domingo K., Grossman D.C., Curry S.J., Davidson K.W., Epling J.W., Garcia F.A., Gillman M.W., Kemper A.R., Krist A.H., US Preventive Services Task Force (2016). Statin use for the primary prevention of cardiovascular disease in adults: US preventive services task force recommendation statement. JAMA.

[175] Oda T., Wu H.C. (1994). Effect of lovastatin on the cytotoxicity of ricin, modeccin, *Pseudomonas* toxin, and diphtheria toxin in brefeldin A-sensitive and -resistant cell lines. Exp. Cell Res..

[176] Gomes A.Q., Ali B.R., Ramalho J.S., Godfrey R.F., Barral D.C., Hume A.N., Seabra M.C. (2003). Membrane targeting of Rab GTPases is influenced by the prenylation motif. Mol. Biol. Cell.

[177] Pfeffer S., Aivazian D. (2004). Targeting Rab GTPases to distinct membrane compartments. Nat. Rev. Mol. Cell Biol..

[178] Holstein S.A., Knapp H.R., Clamon G.H., Murry D.J., Hohl R.J. (2006). Pharmacodynamic effects of high dose lovastatin in subjects with advanced malignancies. Cancer Chemother. Pharmacol..

[179] Schafer W., Stroh A., Berghofer S., Seiler J., Vey M., Kruse M.L., Kern H.F., Klenk H.D., Garten W. (1995). Two independent targeting signals in the cytoplasmic domain determine trans-Golgi network localization and endosomal trafficking of the proprotein convertase furin. EMBO J..

[180] Plaimauer B., Mohr G., Wernhart W., Himmelspach M., Dorner F., Schlokat U. (2001). ‘Shed’ furin: Mapping of the cleavage determinants and identification of its C-terminus. Biochem. J..

[181] Thomas G. (2002). Furin at the cutting edge: from protein traffic to embryogenesis and disease. Nat. Rev. Mol. Cell Biol..

[182] Seidah N.G., Prat A. (2012). The biology and therapeutic targeting of the proprotein convertases. Nat. Rev. Drug Discov..

[183] Couture F., D’Anjou F., Day R. (2011). On the cutting edge of proprotein convertase pharmacology: from molecular concepts to clinical applications. Biomol. Concepts.

[184] De Cicco R.L., Bassi D.E., Benavides F., Conti C.J., Klein-Szanto A.J. (2007). Inhibition of proprotein convertases: approaches to block squamous carcinoma development and progression. Mol. Carcinog..

[185] Sarac M.S., Cameron A., Lindberg I. (2002). The furin inhibitor hexa-d-arginine blocks the activation of *Pseudomonas aeruginosa* exotoxin A in vivo. Infect. Immun..

[186] Sarac M.S., Peinado J.R., Leppla S.H., Lindberg I. (2004). Protection against anthrax toxemia by hexa-d-arginine in vitro and in vivo. Infect. Immun..

[187] Basak A. (2005). Inhibitors of proprotein convertases. J. Mol. Med. (Berl.).

[188] Shayman J.A. (2013). The design and clinical development of inhibitors of glycosphingolipid synthesis: Will invention be the mother of necessity?. Trans. Am. Clin. Climatol. Assoc..

[189] Liu Y.Y., Hill R.A., Li Y.T. (2013). Ceramide glycosylation catalyzed by glucosylceramide synthase and cancer drug resistance. Adv. Cancer Res..

